# Deletion of Pleiotrophin protects against high-fat diet-induced liver metabolic disease, independently of the sexual dimorphism in dietary response

**DOI:** 10.1186/s43556-026-00547-9

**Published:** 2026-08-20

**Authors:** Héctor Cañeque-Rufo, Agata Zuccaro, Marta Inmaculada Sanz-Cuadrado, Federica Ruin, Ángela Olivera-Rodríguez, Javier Pizarro-Delgado, María Limones, Jimena Pita-Santibáñez, María Gracia Sánchez-Alonso, Julio Sevillano, Irma García-Martínez, Ángela M. Valverde, Esther Gramage, Gonzalo Herradón, María del Pilar Ramos-Álvarez

**Affiliations:** 1 Department of Chemistry and Biochemistry, School of Pharmacy, Universidad San Pablo-CEU, CEU Universities, Urbanización Montepríncipe, Boadilla del Monte, 28660 Spain; 2 Department of Health and Pharmaceutical Sciences, School of Pharmacy, Universidad San Pablo-CEU, CEU Universities, Urbanización Montepríncipe, Boadilla del Monte, 28660 Spain; 3 Department of Systems Medicine, University Hospital of Padua, Padua, Italy; 4 Instituto de Investigaciones Biomédicas Sols-Morreale (CSIC-UAM), Madrid, 28029 Spain; 5 Centro de Investigación Biomédica en Red de Diabetes y Enfermedades Metabólicas Asociadas (CIBERdem), Instituto de Salud Carlos III, Madrid, 28029 Spain

**Keywords:** Pleiotrophin, Obesity, Metabolic syndrome, High-fat diet, MASLD, MASH

## Abstract

Obesity is a global health problem linked to the development of metabolic syndrome and comorbidities such as metabolic dysfunction-associated steatotic liver disease (MASLD) and metabolic dysfunction-associated steatohepatitis (MASH). Pleiotrophin (PTN) is a cytokine known for its role in tissue regeneration and energy metabolism. However, its function in hepatic metabolism and its role in MASLD progression remain to be elucidated. Here, we investigated the contribution of PTN to hepatic metabolism using male and female wild-type (*Ptn*^+/+^) and PTN-deficient (*Ptn*^−/−^) mice fed a standard or high-fat diet (HFD) for 6 months, as well as primary hepatocytes and Huh7 cells. *Ptn*-deletion protected both sexes against HFD-induced body weight gain, fasting hyperglycemia, hyperinsulinemia, insulin resistance and metainflammation. Furthermore, PTN deficiency prevented hepatic steatosis, reduced triacylglyceride accumulation and protected against liver fibrosis. Mechanistically, PTN deficiency was associated with increased AMPK activation, reduced ACC abundance and phosphorylation, constitutively low DGAT2 expression and altered AKT signalling. In vitro, PTN directly promoted lipid accumulation and triacylglyceride synthesis in primary hepatocytes and Huh7 cells, supporting a direct role for PTN in regulating hepatocyte lipid metabolism. Our results highlight PTN as a key modulator of hepatic lipid metabolism, systemic inflammation and extracellular matrix remodelling during obesity. These findings identify PTN as a promising therapeutic target for MASLD, MASH and related metabolic disorders, and point to a sexual dimorphism in adaptive metabolic strategies, with females demonstrating a greater degree of protection against the liver-damaging effects of diet-induced obesity.

**Supplementary Information:**

The online version contains supplementary material available at 10.1186/s43556-026-00547-9.

## Introduction

Obesity has become one of the most prevalent diseases worldwide [1]. This chronic disease is linked to metabolic syndrome (MetS) [2], a complex disorder characterised by cardiovascular and metabolic alterations, including hypertension, dyslipidemia, fasting hyperglycemia, and hyperinsulinemia, among others [3]. These alterations lead to systemic inflammation (metainflammation), contributing to diseases such as type 2 diabetes, metabolic dysfunction-associated steatotic liver disease (MASLD), one of the most harmful obesity-associated comorbidities, and even neurodegenerative diseases [4–6]. Excessive lipid accumulation in hepatocytes results in hepatic steatosis [7]. This condition can present as MASLD, including simple fatty infiltration. When fat accumulation triggers lipotoxicity, together with immune-cell infiltration and the release of pro-inflammatory cytokines, the disease progresses to metabolic dysfunction-associated steatohepatitis (MASH) [7–9]. In this context, MetS significantly increases the risk of simple steatosis progressing to MASH, leading to impaired liver function [7, 10].

Several studies reported that premenopausal women have a lower prevalence and severity of liver fibrosis than men, suggesting an estrogen-protective role that declines after menopause [11, 12]. Although several pathways have been proposed, the exact molecular mechanisms underlying this sex-related protection against liver fibrosis are unclear. The resulting liver damage disrupts peripheral metabolism that ultimately affects other organs [10]. Moreover, obesity-associated comorbidities overload health systems and require substantial financial resources, highlighting the need for new therapeutic targets for this disease.

Pleiotrophin (PTN) is a cytokine mainly expressed in the central nervous system [5, 13], with its expression being restricted in adulthood except during tissue regeneration, cell growth and angiogenesis [14–23]. Previous studies from our group have highlighted its role in regulating insulin sensitivity, energy metabolism and thermogenesis [24, 25]. Although PTN is expressed in the adult liver and contributes to liver regeneration [18, 22], its role in hepatic metabolism and MASLD remains unknown. Our recent findings suggest that *Ptn-*deletion may protect against hepatic steatosis, as female mice fed a 45%-HFD (high-fat diet) for three months showed reduced lipid accumulation compared to wild-type counterparts [25]. Whether this effect extends to males and to advanced stages of obesity-associated liver injury, including fibrosis, remains unknown. Therefore, further studies are needed to clarify the role of PTN in hepatic metabolism under physiological and pathological conditions.

In the present study, we investigated the role of endogenous PTN in hepatic metabolism using male and female wild-type (*Ptn*^+/+^) and PTN-deficient (*Ptn*^−/−^) mice subjected to long-term HFD feeding. To complement the in vivo studies and explore the underlying molecular mechanisms, in vitro experiments were performed in primary hepatocytes and Huh7 cells. We demonstrate that *Ptn-*deletion protects against HFD-induced body-weight gain, metainflammation, hepatic steatosis and fibrosis through the modulation of lipid metabolism and insulin-related signalling pathways. Furthermore, our findings reveal sexual dimorphism in the response to HFD, with females exhibiting greater protection against diet-induced liver damage. Overall, this study identifies PTN as a key regulator of liver metabolic homeostasis and highlights its potential as a therapeutic target for MASLD, MASH and other obesity-associated metabolic disorders.

## Results

### *Ptn*-deletion protects against high-fat diet-induced obesity and improves glucose homeostasis

**Fig. 1:**
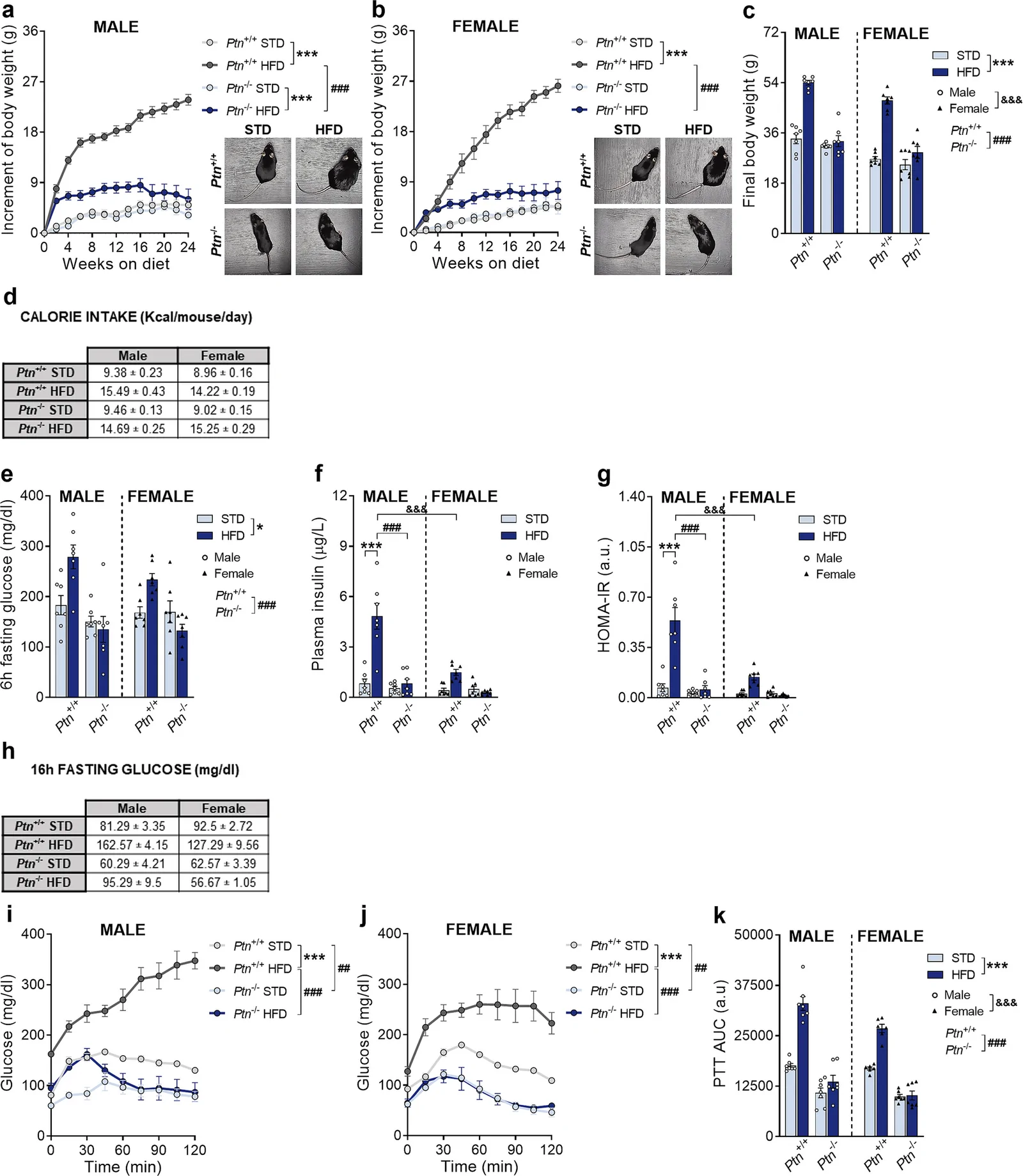
High-fat diet feeding and *Ptn*-deletion effects on body weight and gluconeogenesis from pyruvate. Increment of body weight over time in male (**a**) and female (**b**), final body weight (**c**) and daily calorie intake (**d**) in *Ptn*^+/+^ and *Ptn*^−/−^ mice fed with a STD (18% kcal fat) or HFD (60% kcal fat) for 6 months starting at 3 months of age. Plasma glucose levels after 6 h fasting (**e**), plasma insulin levels (**f**), and the homeostasis model assessment (HOMA) index (**g**). For the gluconeogenesis study, male and female *Ptn*^+/+^ and *Ptn*^−/−^ mice fed a STD or an HFD for 3 months starting at 3 months of age were analysed. Basal glucose after 16 h fasting (**h**); glycemia during the pyruvate tolerance test (PTT; 2 g/kg sodium pyruvate, intraperitoneally) in male (**i**) and female (**j**), and area under the curve (AUC) values from the PTT calculated from the glucose response curves shown in panels i and j (**k**). Data are presented as mean ± SEM (n = 7 animals per group). Differences between diets are represented with: *p < 0.05; ***p < 0.001; between genotypes with: ^##^*p* < 0.01; ^###^*p* < 0.001; and between sexes with: ^&&&^*p* < 0.001

To assess whether *Ptn*-deletion affects body weight (BW) gain in response to an HFD, we monitored the animals (Fig. 1a-c). Although male, but not female, *Ptn*^−/−^ mice initially showed increased BW when fed an HFD, this did not affect the final BW gain. Overall, *Ptn*-deletion protects against HFD-induced BW gain in both sexes independently of food intake (Fig. S1).

We observed that HFD increased 6 h fasting glycemia in control mice, whereas *Ptn*^−/−^ animals were protected against HFD-induced hyperglycemia (Fig. 1e). Regarding plasma insulin, our results indicated that HFD induces hyperinsulinemia in *Ptn*^+/+^ mice, more markedly in male than in female mice, but not in *Ptn*^−/−^ animals (Fig. 1f). Consistently, the HOMA-IR revealed a significant increase in insulin resistance in HFD-*Ptn*^+/+^ mice, again more marked in male than in female animals; no insulin resistance was observed in *Ptn*^−/−^ mice (Fig. 1g).

To evaluate whether *Ptn*-deletion influences hepatic gluconeogenesis and long-fasting glucose levels in response to an HFD, we performed a pyruvate tolerance test (PTT). 16 h fasting glycemia shows a similar profile to that after 6 h fasting (Fig. 1h). Next, PTT was performed. HFD results in a significant rise in glycemia following pyruvate injection, whereas in females this effect tends to normalise over time this is not the case in males, reflecting the insulin resistance observed in these animals. Interestingly, *Ptn*^−/−^ animals showed lower glycemia after pyruvate injection, and this was unaffected by HFD, with females showing the same levels whether they were fed an HFD or STD (Fig. 1i and j). After studying the changes over time in blood glucose after pyruvate injection, the area under the curve (AUC) was calculated, providing a similar interpretation (Fig. 1k). Overall, *Ptn*-deletion is associated with decreased hepatic gluconeogenesis and mitigates its increase induced by HFD, further supporting the protective metabolic role of PTN deficiency.

### *Ptn*, *Mdk* and PTN receptors mRNA in the liver are differentially regulated by sex, genotype and diet

**Fig. 2:**
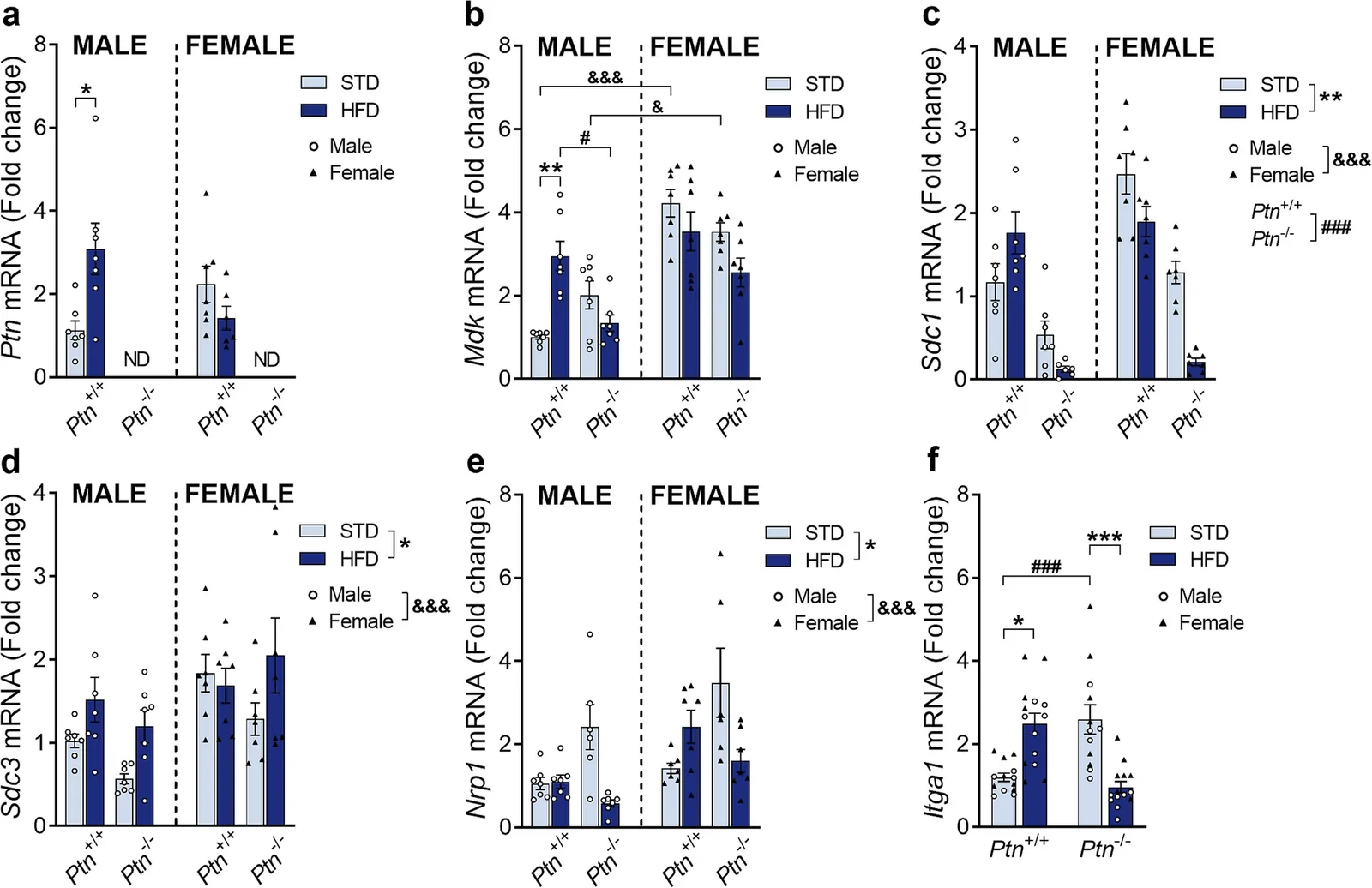
Hepatic mRNA of *Ptn*, *Mdk*, and PTN receptors is modulated by genotype, diet, and sex. Liver samples from 9-month-old male and female *Ptn*^+/+^ and *Ptn*^−/−^ mice were collected after 6 months of feeding with either STD (18% kcal fat) or HFD (60% kcal fat) starting at 3 months of age. Hepatic mRNA levels of pleiotrophin (*Ptn*) (**a**), midkine (*Mdk*) (**b**), syndecan-1 (*Sdc1*) (**c**), syndecan-3 (*Sdc3*) (**d**), neuropilin (*Nrp1*) (**e**) and integrin alpha 1 (*Itga1*) (**f**) were measured by qPCR and normalised to actin (*Actb*) and ribosomal protein L13 (*Rpl13*) mRNA. mRNA expression of anaplastic lymphoma kinase (*Alk*) and protein tyrosine phosphatase receptor type Z1 (*Ptprz1*) was below detection limits. Data are presented as mean ± SEM (n = 7 animals per group; circles correspond to males and triangles correspond to females). Differences between diets are represented with: *p < 0.05; **p < 0.01; ***p < 0.001; between genotypes with: ^#^*p* < 0.05; ^###^*p* < 0.001; and between sexes with: ^&^*p* < 0.05; ^&&&^*p* < 0.001

We examined the mRNA of *Ptn*, midkine (*Mdk*), and some of the best characterised PTN receptors in the liver. HFD significantly increased *Ptn* mRNA only in male *Ptn*^+/+^ mice (Fig. 2a), suggesting that hepatic *Ptn* expression is induced by HFD in a sex-specific manner, which may contribute to differential susceptibility to diet-induced metabolic alterations. Similarly, HFD significantly increased *Mdk* mRNA in male *Ptn*^+/+^ but not in *Ptn*^−/−^ mice (Fig. 2b). Those changes were not found in females, which showed higher *Mdk* mRNA than male mice, suggesting that *Ptn*-deletion modulates *Mdk* expression in a sex- and diet-dependent manner.

We found that syndecans are the most expressed PTN receptor in the mouse liver. Genotype, diet, and sex significantly affected syndecan-1 (*Sdc1*) mRNA (Fig. 2c), whereas diet and sex, but not genotype, significantly modified syndecan-3 (*Sdc3*) mRNA (Fig. 2d). We found a low expression of neuropilin (*Nrp1*) and integrin alpha 1 (*Itga1*) (analysed using tenfold more concentrated cDNA than for syndecans) (Fig. 2e and f), which was modulated in a genotype-specific manner by HFD. The integrin beta 3 (*Itgb3*), low-density lipoprotein receptor-related protein 1 (*Lrp1*), nucleolin (*Ncl*), ALK receptor tyrosine kinase (*Alk*) and the protein tyrosine phosphatase receptor type Z1 (*Ptprz1*) mRNA were below detection limits (data not shown). These findings indicate that the expression of PTN receptors is sensitive to both sex and diet, highlighting potential mechanisms through which PTN signalling could differentially regulate hepatic metabolism in males and females.

### *Ptn*-deficiency protects against high-fat diet-induced systemic inflammation and liver damage

**Fig. 3:**
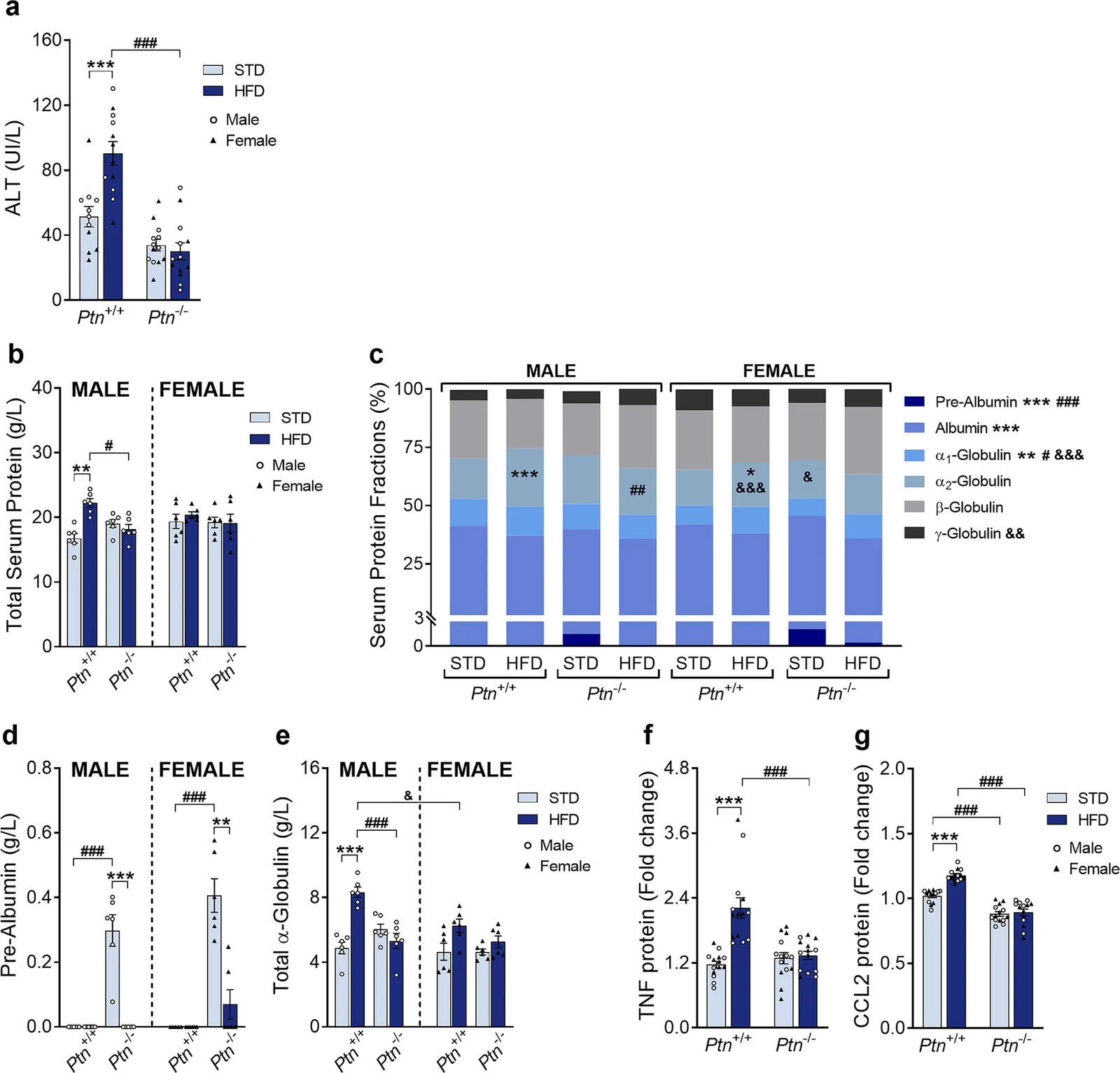
High-fat diet feeding and *Ptn*-deletion effects on serum proteins, circulating proinflammatory molecules and ALT. Male and female *Ptn*^+/+^ and *Ptn*^−/−^ mice were fed a STD (18% kcal fat) or an HFD (60% kcal fat) for 6 months. Plasma alanine aminotransferase (ALT) levels (**a**). Total serum protein concentration determined by the BCA assay (**b**). Percentage distribution of serum protein fractions obtained by agarose gel electrophoresis (**c**). Serum pre-albumin (**d**) and total α-globulin (**e**) levels quantified from electrophoretic profiles. Circulating tumour necrosis factor (TNF) (**f**) and C–C motif chemokine 2 (CCL2) (**g**) measured using the Olink® system. Data are presented as mean ± SEM (n = 6–7 animals per group; circles correspond to males and triangles correspond to females). Differences between diets are represented with: *p < 0.05; **p < 0.01; ***p < 0.001; between genotypes with: ^#^*p* < 0.05; ^##^*p* < 0.01; ^###^*p* < 0.001; and between sexes with: ^&^*p* < 0.05; ^&&^*p* < 0.01; ^&&&^*p* < 0.001

Circulating alanine aminotransferase (ALT) activity was measured as a marker of hepatocellular injury (Fig. 3a). Statistical analysis revealed that genotype and diet, but not sex, significantly affected ALT activity. To further explore the effect of genotype and diet, we employed two-way ANOVA excluding sex, finding a significant increase in circulating ALT activity only in HFD-*Ptn*^+/+^ animals. These results indicate that HFD is accompanied by liver damage, which does not happen in *Ptn*^−/−^ mice.

Then, we analysed the circulating proteins as markers of liver function. HFD significantly increased the protein concentration only in male *Ptn*^+/+^ mice (Fig. 3b). Also, we studied the relative percentage of the protein fractions in each group (Fig. 3c). In brief, HFD increased α_1_-globulin and α_2_-globulin and decreased the albumin relative percentages only in *Ptn*^+/+^ animals. Furthermore, the γ-globulin relative percentage was increased in female mice.

We observed similar results when the concentration of each protein fraction was calculated (Fig. 3d, e and Fig. S2). Overall, we found that HFD-*Ptn*^+/+^ animals showed an increase in α-globulin, an effect that does not occur in HFD-*Ptn*^−/−^ animals. These findings highlight a systemic inflammatory protective effect of PTN deficiency. The presence of pre-albumin was particularly striking, as it was observed only in *Ptn*^−/−^ animals and decreased with HFD.

To complete the analysis of the effects of HFD and *Ptn*-deletion on metainflammation, proinflammatory plasma molecules were analysed. HFD significantly increases tumour necrosis factor (TNF) (Fig. 3f) and C–C motif chemokine 2 (CCL2) (Fig. 3g) in *Ptn*^+/+^ but not in *Ptn*^−/−^ animals, which even have lower plasma levels of CCL2 at baseline. Overall, *Ptn-*deletion limits HFD-induced systemic inflammation and liver damage, which may explain the improved metabolic profile observed in these animals.

### *Ptn*-deficiency prevents the development of hepatic steatosis induced by high-fat diet

**Fig. 4:**
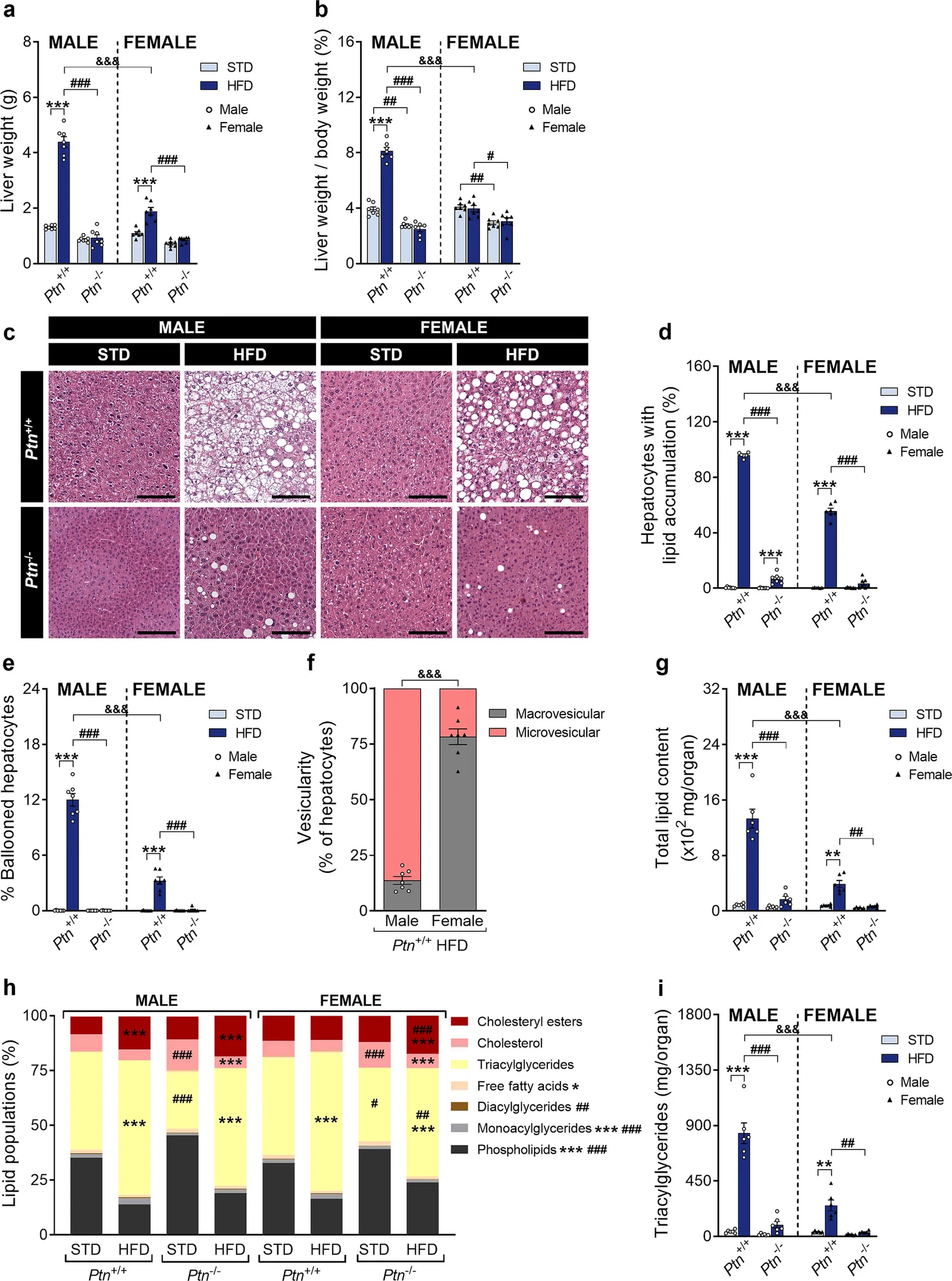
High-fat diet feeding and *Ptn*-deletion effects on liver weight and steatosis. Male and female *Ptn*^+/+^ and *Ptn*^−/−^ mice were fed a STD (18% kcal fat) or an HFD (60% kcal fat) for 6 months starting at 3 months of age. Liver weight (**a**) and relative liver weight (**b**). Representative haematoxylin and eosin (H&E) staining of formalin-fixed paraffin-embedded liver sections showing the degree of hepatic steatosis (**c**). Percentage of hepatocytes with lipid accumulation (**d**) and percentage of ballooned hepatocytes (**e**) quantified from H&E-stained sections. Characterisation of lipid vesicularity (macrovesicular vs. microvesicular) in groups displaying steatosis (**f**). Total hepatic lipid content determined by the Folch extraction method (**g**). Distribution of lipid fractions separated by thin-layer chromatography (**h**). Triacylglycerides (TAG) content in liver lipid extracts (**i**). Data are presented as mean ± SEM (n = 7 animals per group; circles correspond to males and triangles correspond to females). Differences between diets are represented with: *p < 0.05; **p < 0.01; ***p < 0.001; between genotypes with: ^#^*p* < 0.05; ^##^*p* < 0.01; ^###^*p* < 0.001; and between sexes with: ^&&&^*p* < 0.001. Scale bar 200 µm

We analysed whether *Ptn-*deletion protects against HFD-induced lipid accumulation in the liver. HFD produced a significant increase in liver weight only in *Ptn*^+/+^ mice, which was more pronounced in male than in female animals (Fig. 4a). When normalising liver weight to BW, we found that it was increased only in male HFD-*Ptn*^+/+^ animals. In addition, *Ptn*^−/−^ animals, regardless of diet, had a lower relative liver weight than *Ptn*^+/+^ animals (Fig. 4b).

H&E staining allowed the study of the degree of hepatic steatosis (Fig. 4c). The percentage of hepatocytes with lipid accumulation (Fig. 4d) and of ballooned hepatocytes (Fig. 4e) indicate that only HFD-*Ptn*^+/+^ animals showed a clear state of hepatic steatosis. Following this observation, the vesicularity in those groups with hepatic steatosis was analysed (Fig. 4f). We found that after feeding HFD, male *Ptn*^+/+^ animals had a higher percentage of microvesicularity compared to female *Ptn*^+/+^ mice, which had a higher percentage of macrovesicularity (Fig. 4f). This sex-dependent pattern suggests that males are more susceptible to severe hepatocellular damage, whereas females develop a slower, less damaging form of steatosis. Conversely, the absence of PTN confers strong protection against HFD-induced hepatic steatosis and hepatomegaly, supporting the role of PTN as a positive regulator of hepatic lipid accumulation.

Following the observed results, we analysed the lipid content to determine whether *Ptn-*deletion affects specific lipid populations involved in steatosis progression. Our results revealed that HFD leads to a significant increase in hepatic lipids in *Ptn*^+/+^ animals, which is more pronounced in males than in females. However, *Ptn*^−/−^ animals are protected against HFD-induced hepatic lipid accumulation, regardless of sex (Fig. 4g and Fig. S3a). We then tested whether the different types of lipids change in the same way (Fig. 4h). In summary, HFD intake increased the percentage of cholesteryl esters, triacylglycerides (TAG) and monoacylglycerides (MAG), but decreased the percentage of phospholipids and free fatty acids in *Ptn*^+/+^ animals. Moreover, the *Ptn-*deletion was associated with a decrease in the percentage of all acylglycerides, while increasing cholesterol and phospholipids. HFD partially restored these values to levels like those observed in *Ptn*^+/+^ mice.

Finally, we studied each of the lipid species individually to determine the total lipid content in the liver (Fig. S3). HFD significantly increased the content of all lipid populations in *Ptn*^+/+^ animals, which was more pronounced in male than in female mice. However, *Ptn-*deletion protected against these HFD-induced alterations. Altogether, these findings reinforce the concept that PTN plays an important role in regulating hepatic lipid accumulation and composition in response to HFD-stress.

### *Ptn*-deletion alters proteins involved in hepatic lipid metabolism, protecting against HFD-induced metabolic disruptions

**Fig. 5:**
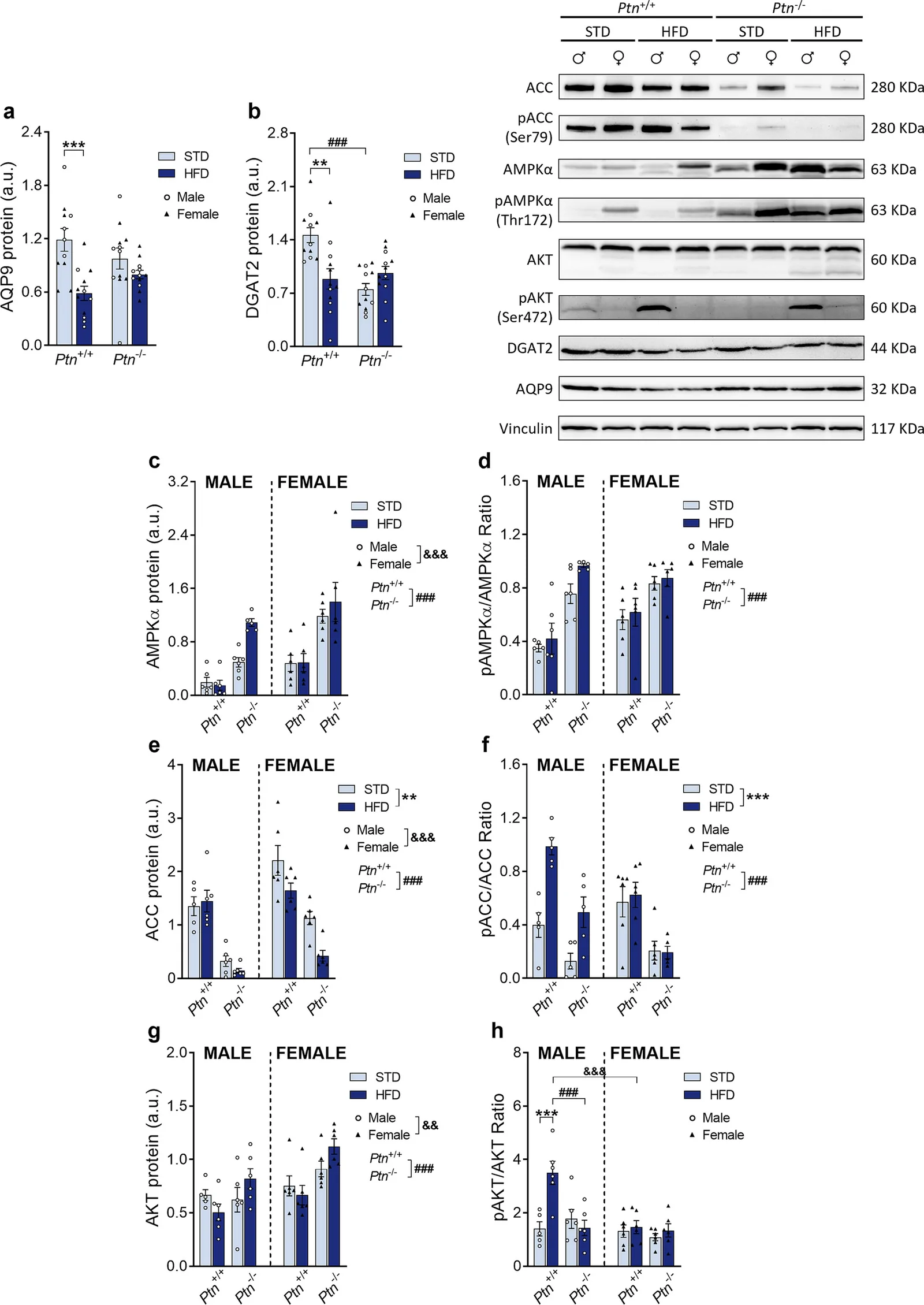
High-fat diet and *Ptn*-deletion effects on the proteins involved in lipid synthesis in the liver. Liver samples from 9-month-old male and female *Ptn*^+/+^ and *Ptn*^−/−^ mice were collected after 6 months of feeding with either STD (18% kcal fat) or HFD (60% kcal fat) starting at 3 months of age. Hepatic protein levels of aquaporin 9 (AQP9) (**a**), diacylglycerol O-acyltransferase 2 (DGAT2) (**b**), AMP-activated protein kinase α (AMPKα) (**c**), pAMPKα(Thr172)/AMPKα ratio (**d**), acetyl-CoA carboxylase (ACC) (**e**), pACC(Ser79)/ACC ratio (**f**), protein kinase B (AKT) (**g**), and pAKT(Ser472)/AKT ratio (**h**) were determined by Western blot analysis. Vinculin was used as a loading control. Data are presented as mean ± SEM (n = 6 animals per group; circles correspond to males and triangles correspond to females). Differences between diets are represented with: **p < 0.01; ***p < 0.001; between genotypes with: ^###^*p* < 0.001; and between sexes with: ^&&^*p* < 0.01; ^&&&^*p* < 0.001

We analysed the amount and phosphorylation of key lipid-synthesis proteins to understand how the observed lipid changes occurred (Fig. 5). For AQP9 (Fig. 5a) and DGAT2 (Fig. 5b), statistical analysis showed no effects of the sex variable, so we excluded it to further explore the effect of genotype and diet. Our results indicated that *Ptn*^−/−^ animals were protected against the HFD-induced decrease in AQP9 and DGAT2 observed in *Ptn*^+/+^ animals. In addition, *Ptn*^−/−^ mice had low DGAT2, which was not affected by HFD.

Finally, we studied the AMPKα, ACC, and AKT proteins and their phosphorylated forms (Fig. 5c-h and Fig. S4). We observed that females had higher AMPKα, ACC, and AKT protein levels than male animals, and that HFD decreased ACC protein in females but not in male mice. Moreover, we found that in the livers of *Ptn*^−/−^ mice, there was a significant increase in AKT and AMPKα, alongside a marked decrease in ACC. When studying AMPKα, ACC, and AKT phosphorylation, we found that *Ptn*^−/−^ animals show a significant increase in pAMPKα/AMPKα in contrast to a decrease in pACC/ACC ratio when compared to *Ptn*^+/+^ mice. In addition, an increase in pAKT/AKT ratio was found only in HFD-*Ptn*^+/+^. This pattern suggests that *Ptn*-deletion activates the AMPKα pathway, promoting fatty acid oxidation and energy balance while inhibiting lipogenesis through reduced ACC amount and activation, as well as AKT activation. Overall, these data suggest that the absence of PTN preserves metabolic flexibility and protects the liver from HFD-induced lipotoxicity.

### *Ptn*-deletion decreases collagen in the tunica adventitia of blood vessels and protects against the development of HFD-induced liver fibrosis

**Fig. 6:**
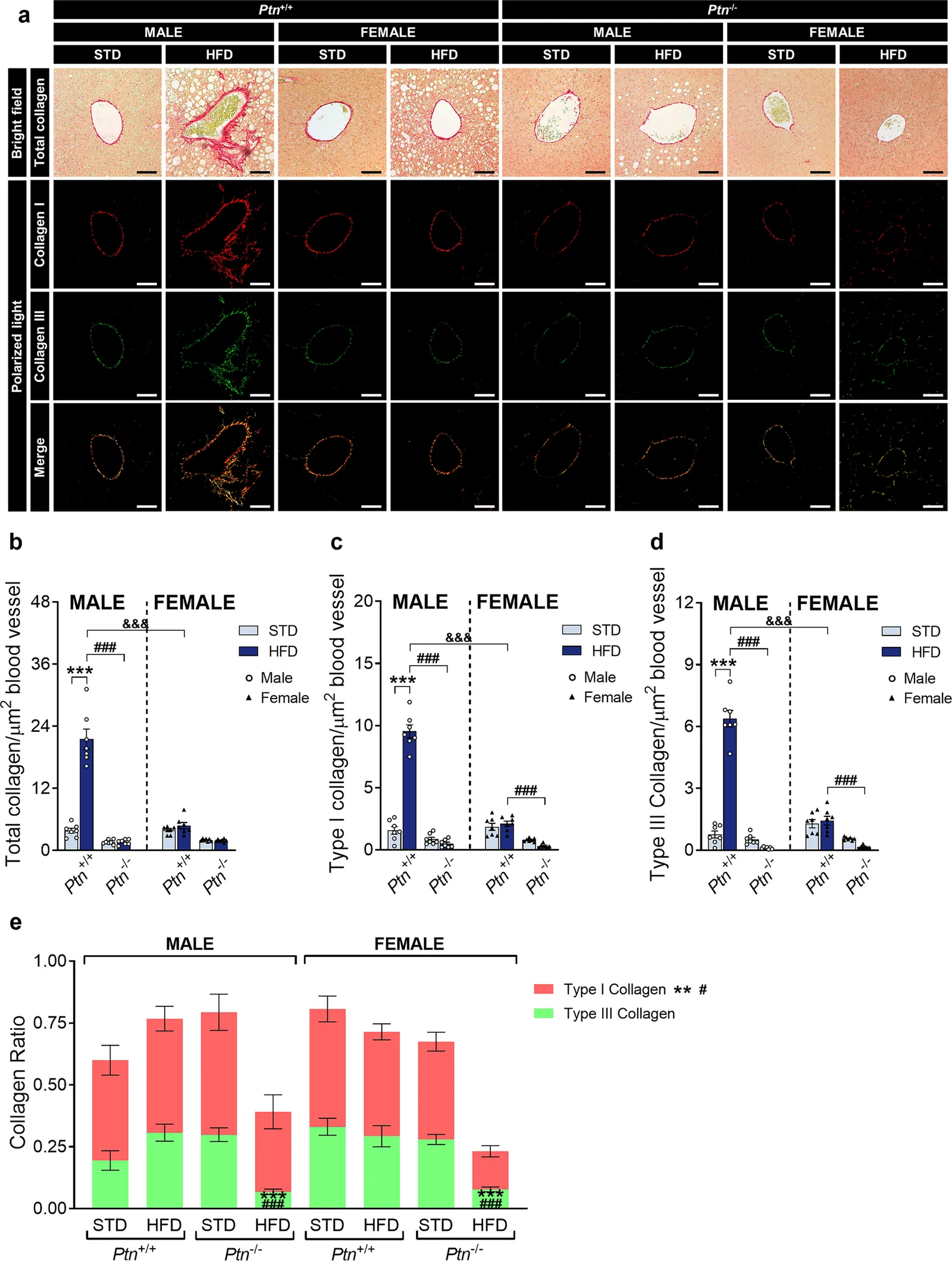
High-fat diet feeding and *Ptn*-deletion effects on liver fibrosis. Male and female *Ptn*^+/+^ and *Ptn*^−/−^ mice were fed a STD (18% kcal fat) or an HFD (60% kcal fat) for 6 months starting at 3 months of age. Representative images of Picrosirius Red-stained formalin-fixed paraffin-embedded liver sections showing collagen deposition (**a**). Quantification of the marked area per µm^2^ of blood vessel for total collagen (**b**), type I collagen (**c**), and type III collagen (**d**), as determined by bright-field and polarised light microscopy. Ratio of type I and type III collagen relative to total collagen content (**e**). Data are presented as mean ± SEM (n = 7 animals per group). Differences between diets are represented with: **p < 0.01; ***p < 0.001; between genotypes with: ^#^*p* < 0.05; ^###^*p* < 0.001; and between sexes with: ^&&&^*p* < 0.001. Scale bar 200 µm

Given the role of PTN in tissue remodelling and fibrogenesis, we determined whether its deletion modulates the development of HFD-induced hepatic fibrosis by analysing total, type I and type III collagen of the tunica adventitia of blood vessels using picrosirius red collagen staining (Fig. 6). Our data revealed that HFD favoured liver fibrosis, increasing the total, type I, and type III collagen (Fig. 6b-d), and their relative proportions, only in male *Ptn*^+/+^ animals (Fig. 6e). *Ptn*-deletion not only protected against this effect of HFD, but also reduced collagen in the tunica adventitia of blood vessels. These results indicate that PTN may contribute to HFD-induced hepatic fibrogenesis, likely favouring extracellular matrix (ECM) deposition and vascular remodelling. Its absence, therefore, confers structural protection to the liver, maintaining a more quiescent ECM profile even under metabolic stress.

### PTN induces triacylglyceride deposition in hepatocytes and reverses the metabolic phenotype of PTN-deficient liver cells

**Fig. 7:**
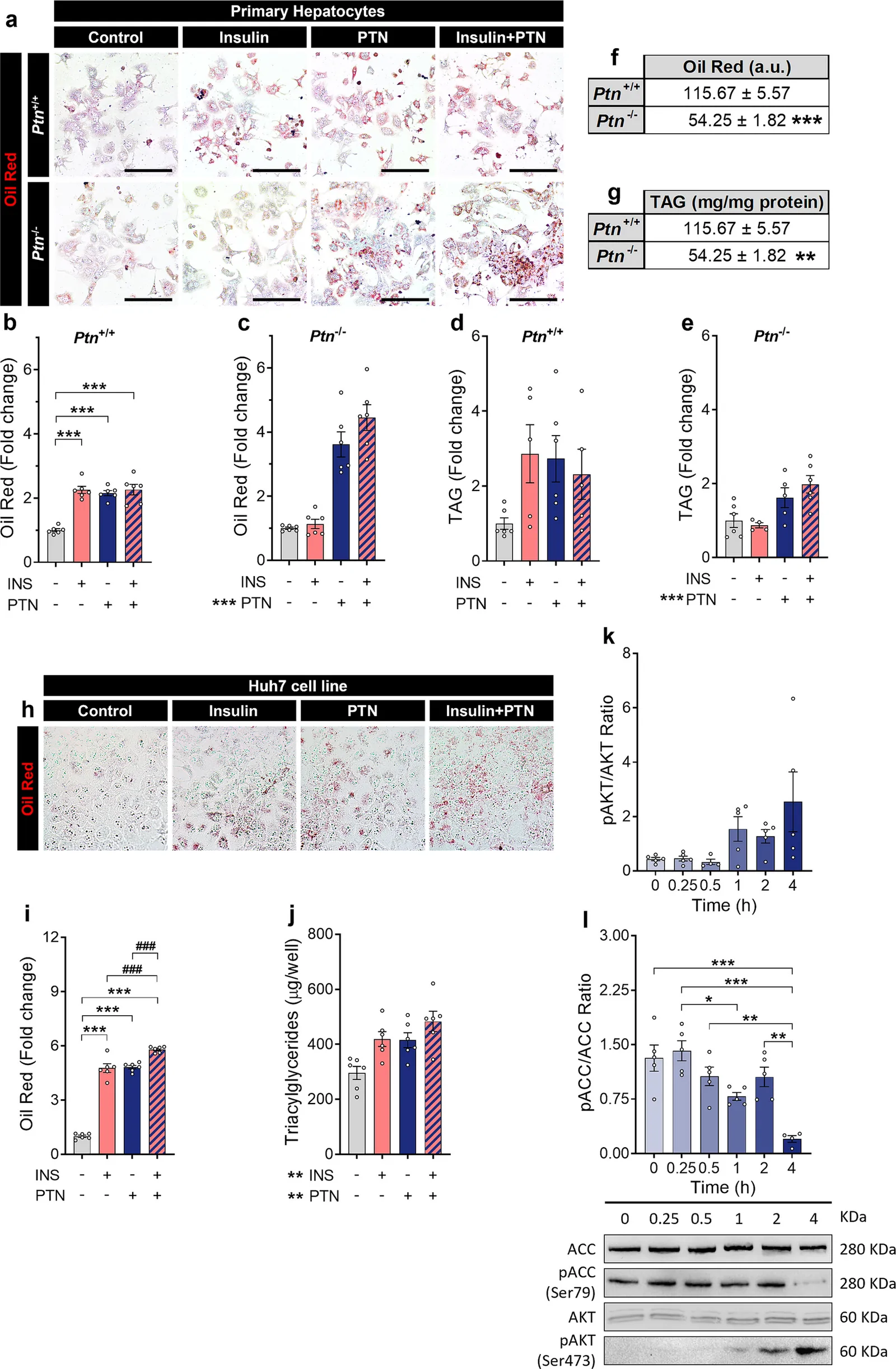
Insulin and PTN effects on lipid accumulation in primary hepatocytes and Huh7 cell line. *Ptn*^+/+^ and *Ptn*^−/−^ primary hepatocytes were cultured in attachment medium for 24 h. For treatments, cells were incubated for 48 h with 10 nM human recombinant insulin, 0.5 µg/mL recombinant PTN, or both in low-glucose DMEM and serum-starved (0% FBS). Huh7 cells were cultured in high-glucose DMEM supplemented with 10% fetal bovine serum and 1% penicillin/streptomycin. For treatments, cells were serum-starved (3% FBS) and incubated for 48 h with 10 nM human recombinant insulin, 0.5 µg/mL recombinant PTN, or both. Representative Oil Red O staining of intracellular lipid droplets (**a** and **h**). Quantification of lipid accumulation expressed as fold-change relative to untreated *Ptn*^+/+^ (**b**), *Ptn*^−/−^ primary hepatocytes (**c**), and Huh7 cell line (**i**). Analysis was performed using *Fiji* software (NIH, Bethesda, MD, USA, version 1.50f). Images (20X objective) from each well were acquired to estimate the area stained with OR and normalised by cell number. Quantification of triacylglycerides (TAG) content (determined by the glycerol-3-phosphate/peroxidase colorimetric method) in *Ptn*^+/+^ (**d**), *Ptn*^−/−^ primary hepatocytes (**e**), and Huh7 cell line (**j**). Tables showing the absolute values of the control groups for Oil Red O quantification (**f**) and triglyceride measurements (**g**). Phospho-AKT (Ser473) (pAKT-Ser473)/AKT ratio (**k**) and phospho-acetyl-CoA carboxylase (Ser79) (pACC)/ACC ratio (**l**) measured by Western blot analysis after Huh7 cell line PTN stimulation for the indicated time points. Data are presented as mean ± SEM (n = 5–6 per group). The treatment effects are represented with: **p < 0.01; ***p < 0.001. Differences between treatments are represented with: ^###^*p* < 0.001. Differences between time points are represented with: *p < 0.05; **p < 0.01; ***p < 0.001. Scale bar 200 µm

To determine whether PTN directly regulates lipid metabolism in hepatocytes, we studied the effect of PTN and insulin on *Ptn*^+/+^ and *Ptn*^−/−^ primary hepatocytes. We found that insulin and PTN significantly increase the lipid content in *Ptn*^+/+^ hepatocytes, but only PTN did so in *Ptn*^−/−^ cells (Fig. 7b and c). In addition, PTN significantly increased the TAG accumulation in *Ptn*^−/−^ cells (Fig. 7e). It is important to highlight that *Ptn*^−/−^ primary hepatocytes started from significantly lower values of oil red (OR) staining and TAG concentration (Fig. 7f and g).

To determine whether this phenotype also occurs in a human cell model, Huh7 cells were used (Fig. 7h). Consistent with the results in primary hepatocytes, PTN treatment increased lipid and TAG accumulation to levels comparable to those induced by insulin (Fig. 7i and j). Interestingly, insulin treatment did not induce lipid accumulation in *Ptn*^−/−^ primary hepatocytes, but PTN alone and combined with insulin significantly increased lipid accumulation.

Then, we analysed the effect of PTN on the key lipid-synthesis enzymes in Huh7 cells. PTN significantly increased the pAKT/AKT ratio while decreasing the pACC/ACC ratio (Fig. 7k and l), suggesting that PTN promotes lipogenesis through activation of the AKT pathway and reduction in ACC inhibitory phosphorylation, thereby facilitating lipid synthesis in hepatocytes.

Analysis of PTN receptors expression in Huh7 cells (Fig. S5) revealed, as in primary hepatocytes, that *ALK* and *PTPRZ1* mRNA levels were below detection limits (data not shown). Furthermore, we found that insulin significantly increased the *ITGAV*, *LRP1*, *NRP1* and *SDC1* expression levels.

## Discussion

Obesity and its comorbidities, including MetS and MASLD, are major global health challenges [1–3, 10, 26]. Chronic inflammation and metabolic imbalance are central elements that accelerate liver damage and progression to severe conditions such as MASH [7–9]. In this context, PTN has emerged as a potential regulator of liver homeostasis, although its role in obesity-related metabolic alterations remains insufficiently explored [25].

As expected, HFD induced obesity in *Ptn*^+/+^ mice, whereas *Ptn*^−/−^ animals did not gain weight even after 6 months on a 60%-HFD, confirming previous results in female mice with a less severe dietary treatment [5, 25]. Our study also demonstrates that this protective effect occurs in both sexes. Since food intake was similar among groups, we can hypothesise that *Ptn-*deletion protects against HFD-induced BW gain.

As expected, HFD-fed control mice developed fasting hyperglycemia, hyperinsulinemia and increased HOMA-IR, indicative of insulin resistance. These alterations were more pronounced in males, consistent with their greater susceptibility to metabolic and hepatic damage. In contrast, *Ptn*^−/−^ mice maintained slightly lower fasting glycemia and did not develop HFD-induced hyperinsulinemia or insulin resistance, indicating that PTN may be required for the development of HFD-induced insulin resistance. Obesity is closely associated with liver damage and metabolic dysfunction. Our results suggest that obesity is associated with increased gluconeogenesis, consistent with hepatic insulin resistance [27, 28]. In addition, we observed a sexual dimorphism, with females showing a greater capacity to normalise glycemia over time than males, who displayed sustained hyperglycemia. This suggests an estrogen-mediated protective effect that may attenuate the metabolic dysfunction associated with insulin resistance [29]. By contrast, *Ptn*^−/−^ mice had an opposite metabolic profile, with low basal glycemia and reduced gluconeogenesis, suggesting that PTN may act, directly or indirectly, as a positive regulator of hepatic gluconeogenesis. Since hepatic gluconeogenesis is a major determinant of fasting glycemia, the protection observed in *Ptn*^−/−^ mice aligns with their reduced gluconeogenic capacity and enhanced insulin-mediated suppression of glucose production. This interaction between PTN and insulin in the modulation of hepatic gluconeogenesis may involve the modulation of AKT phosphorylation, as discussed below.

Our results also indicate hepatic damage and metainflammation in *Ptn*^+/+^ animals fed an HFD, as evidenced by the increase in ALT activity, a marker of liver injury, and total α-globulin fractions and a decrease in albumin, indicative of inflammation. This HFD-induced inflammation is less pronounced in female *Ptn*^+/+^ mice, indicating delayed liver damage. Liver damage in *Ptn*^+/+^ animals may trigger a self-perpetuating vicious cycle in which metabolic dysfunction sustains inflammation and alterations in other organs. This is relevant given the fundamental role of the liver in maintaining whole-body homeostasis, as damage to this organ can have detrimental consequences. *Ptn*^−/−^ animals fed an HFD did not show these alterations, suggesting a protective role of *Ptn-*deletion. The analysis of circulating TNF and CCL2 confirms the HFD-induced systemic inflammation, which was absent in *Ptn*^−/−^ animals, consistent with their reduced basal inflammatory state. These results align with previous studies reporting that endogenous PTN modulates systemic TNFα levels under both basal and LPS-induced inflammation [30], and suggest that PTN may be required to trigger the systemic inflammatory response. Consistently, HFD-feeding significantly increased plasma ALT in *Ptn*^+/+^ mice, indicating diet-induced liver damage, whereas *Ptn*^−/−^ mice were protected from this effect. Collectively, our data demonstrate that *Ptn-*deletion protects against HFD-induced liver damage.

The elevated pre-albumin, a transport protein for thyroid hormones [31], in *Ptn*^−/−^ mice suggests a more rapid and efficient transport of thyroid hormones to target tissues. This may explain the decrease in circulating thyroxine that we have previously found in this animal model, likely due to increased metabolism and uptake by major target organs [5, 24].

In the context of MASLD, we observed that HFD led to a marked increase in liver weight in male *Ptn*^+/+^ animals. This alteration is likely due to lipid accumulation in both sexes and the associated hepatic inflammation in males. In contrast, *Ptn*^−/−^ mice exhibited smaller livers regardless of diet, possibly related to the mitogenic role of PTN. Histology confirmed that HFD induced hepatic steatosis in *Ptn*^+/+^ animals, particularly in males, which had increased lipid accumulation and ballooned hepatocytes, a marker of hepatocyte degeneration. Moreover, males and females showed distinct steatosis patterns, with predominantly microvesicular and macrovesicular lipid accumulation, respectively, supporting a sex-dependent response to HFD. Hepatic lipid analysis revealed that HFD increased total lipid classes and content, again more markedly in male *Ptn*^+/+^ mice. Remarkably, *Ptn*^−/−^ mice did not show signs of steatosis or lipid accumulation despite the prolonged duration and the high-fat content of the diet. Lipid-species profiling revealed that *Ptn-*deletion reduced the proportion of all acylglycerides, with TAG being the major species, while increasing cholesterol and phospholipids, likely reflecting changes in key enzymes of lipid synthesis. The high lipid content and TAG proportion in the liver of *Ptn*^+/+^ animals may trigger a compensatory downregulation of AQP9 and DGAT2 to rebalance lipid ratios. In contrast, *Ptn*^−/−^ animals have a constitutive decrease in DGAT2, a key protein for TAG synthesis, suggesting that PTN may influence hepatic lipid synthesis by modulating DGAT2 expression or activity. Reducing or inhibiting this enzyme reduces hepatic fat and protects against HFD-induced steatosis [32, 33]. In this regard, we found a drastic reduction in ACC, the rate-limiting enzyme of lipogenesis, supporting reduced fatty acid synthesis in the liver of these animals. Although HFD was associated with increased inhibitory phosphorylation of ACC, probably as a compensatory mechanism to excess hepatic lipids in males, this was not observed in females, regardless of the diet. Notably, *Ptn*^−/−^ animals have enhanced AMPKα protein and activation, evidenced by a high pAMPKα/AMPKα ratio, together with reduced ACC protein and pACC/ACC ratio, supporting a shift toward reduced hepatic lipogenesis and promoting fatty acid oxidation [34]. This is further supported by the increased OXPHOS proteins that we found in 3-month-deficient *Ptn*^−/−^ mice (unpublished results). The increased AMPKα activity in *Ptn*^−/−^ mice is consistent with their resistance to steatosis. The lower pACC/ACC ratio in *Ptn*^−/−^ mice could reflect an apparent disengagement of the AMPKα-ACC axis. However, this more likely reflects the drastically reduced ACC protein levels, which may mask the degree of its phosphorylation and inhibition. This suggests a metabolic reprogramming that downregulates lipogenesis at both transcriptional and post-translational levels.

We also found that AKT activation only occurs in HFD-fed *Ptn*^+/+^ animals, but not in *Ptn*^−/−^ mice. PTN is a ligand of the syndecan receptors [35, 36], which trigger phosphorylation and activation of AKT [37–39], promoting lipid synthesis in hepatocytes. This result aligns with the higher *Ptn* expression in control male mice after HFD. This differential regulation of AMPKα, ACC and AKT, together with the constitutive downregulation of DGAT2 in the *Ptn*^−/−^ animals, may explain their protection against lipid accumulation and steatosis. It also points to a sexual dimorphism in adaptive metabolic strategies, with females being more resistant to HFD-induced hepatic lipid accumulation.

The expression of *Ptn* and *Mdk* in the liver*,* two homologous and frequently co-regulated cytokines, has been poorly characterised. We found that both are expressed in the liver, with females showing higher *Mdk* expression than males. Interestingly, *Ptn* was overexpressed under HFD only in the male *Ptn*^+/+^ mice, consistent with the higher degree of steatosis and inflammation. Together with the protection observed in *Ptn*^−/−^ animals, these findings suggest that basal PTN contributes to the HFD-induced liver damage, whereas the lack of a similar response in females may reflect their enhanced metabolic resilience, as previously suggested.

In vitro results, in both mouse primary hepatocytes and the human Huh7 cell line, support the role of PTN in lipid synthesis, as PTN induces an increase in TAG and lipid accumulation. Interestingly, although primary hepatocytes from *Ptn*^−/−^ mice have lower basal lipid content than those from *Ptn*^+/+^ animals, as we found in the whole liver, PTN treatment increased lipid accumulation to levels comparable to or even higher than those found in *Ptn*^+/+^ hepatocytes. Interestingly, insulin stimulation did not alter lipid or TAG accumulation in PTN-deficient hepatocytes. We hypothesise that this lack of response is due to decreased ACC expression rather than insulin resistance (unpublished preliminary results). Furthermore, PTN treatment for 48 h may, directly or indirectly, restore ACC expression and, consequently, normalise lipogenic activity. The mechanism by which PTN modulates ACC expression will be the subject of future studies.

We also found that PTN treatments result in decreased ACC phosphorylation, favouring the active form of the enzyme, and higher AKT phosphorylation. This mechanism integrates well with that observed in *Ptn*^−/−^ animals, where PTN absence disrupts these pathways, protecting against hepatic steatosis. This differential expression may play a significant role in the hepatic response to diet.

These alterations lead to a progressive state of liver damage and fibrosis, or MASH [7, 10], which involves not only lipid accumulation but also ECM remodelling, particularly in the tunica adventitia of the blood vessels [40, 41], as we found in male *Ptn*^+/+^ mice fed with HFD. The increase in total collagen was accompanied by higher proportions of type I and III collagens, both closely associated with the structural deterioration characteristic of advanced disease [41]. In contrast, *Ptn-*deletion protected against HFD-induced liver fibrosis, reducing total collagen and ratios of collagen types I and III in the tunica adventitia of blood vessels. This suggests that the PTN absence interferes with pathways involved in ECM synthesis and remodelling, limiting the progression to fibrosis.

The observed sex-specific differences further highlight the influence of sex on susceptibility to HFD-induced liver damage. In this context, the relationship between PTN and ACC seems to play a key role. Other studies suggest that ACC inhibition significantly reduces fibrosis in various MASH models [42]. Accordingly, PTN may increase collagen production and promote HFD-induced liver fibrosis through ACC activation. This is because ACC activity may contribute to lipotoxicity, promoting inflammatory signals and the activation of hepatic stellate cells (HSCs), leading to fibrosis [43]. In the absence of PTN, total ACC, and therefore its activity, are reduced. This, together with lower inflammatory signals, may reduce collagen production through HSCs activation. This is consistent with the protection observed in this animal model and the lower levels of collagen under physiological conditions.

Despite these findings, this study has some limitations. Demonstrating a direct link between PTN and these metabolic pathways would require causal validation using specific inhibitors or other functional approaches. In addition, exploring the molecular mechanisms underlying the observed sex-specific differences would be valuable, as it could provide new insights into how PTN effects are regulated in a sex-dependent manner.

**Fig. 8:**
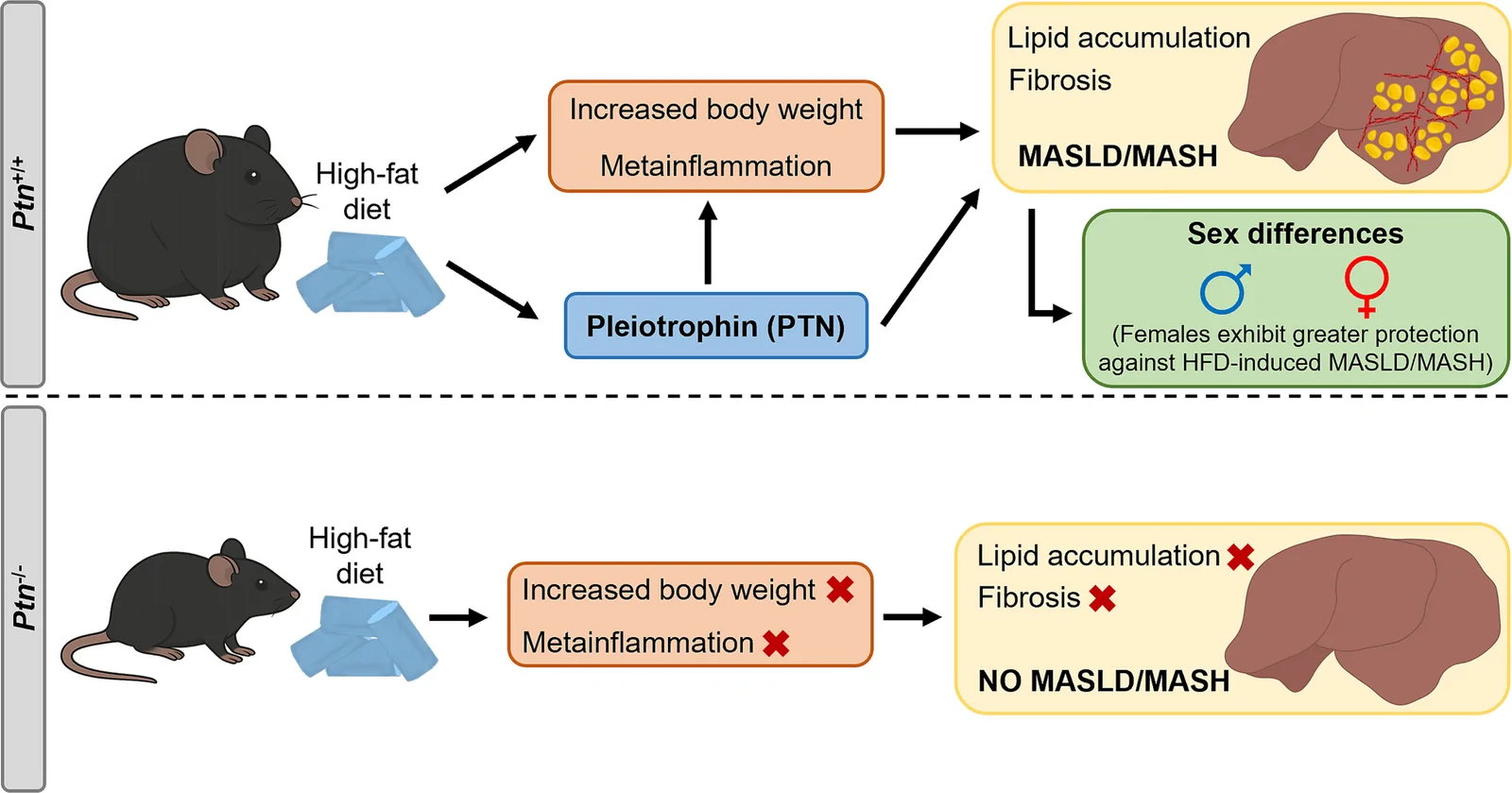
A schematic summary of the results obtained in this study

Overall, this study is relevant given the increasing global prevalence of obesity and MetS, key factors in chronic diseases such as MASLD and MASH. It identifies PTN as a key regulator in metabolic, inflammatory and liver remodelling processes linked to obesity (Fig. 8). *Ptn-*deletion protects against HFD-induced weight gain, systemic inflammation, hepatic lipid accumulation and the development of steatosis and liver fibrosis. Moreover, our study reveals a clear sexual dimorphism in metabolic adaptations to HFD, with females exhibiting greater protection against diet-induced liver damage (Table S4 summarises the overall results). Altogether, these findings contribute to our understanding of the underlying molecular mechanisms and highlight PTN as a promising therapeutic target to address the global problem of these metabolic diseases.

## Materials and methods

### Animals and samples collection

*Ptn*^−/−^ mice were generated as previously described [44]. Male and female C57BL/6 J *Ptn*^+/+^ and *Ptn*^−/−^ mice were randomly divided and housed specific pathogen-free room at 22 ± 1ºC with 12 h light/dark cycles, with free access to water and fed ad libitum with standard chow diet (STD, 18 kcal% fat, 58 kcal% carbohydrates, and 24% kcal protein; 3.1 kcal·g^−1^) or an HFD (D12492, 60 kcal% fat, 20 kcal% carbohydrates, and 20% kcal protein; 5.24 kcal·g^−1^). Dietary intervention started at 3 months of age and was maintained for 6 months. All procedures complied with the European Union Laboratory Animal Care Rules (2010/63/EU directive) and were approved by the Animal Research Committee of CEU San Pablo University and the Comunidad de Madrid (PROEX 140.3/22).

After 6 h fasting, 9-month-old mice (n = 7/group) were sacrificed by decapitation. Liver samples were collected and post-fixed in 4% paraformaldehyde (PFA) for 48 h at 4ºC for histological analysis. Fixed livers were dehydrated, embedded in paraffin, and sectioned at 3 μm thickness using a microtome (Leica Biosystems, Spain). For molecular studies, the livers were collected and stored at −80 ºC. Blood samples were collected in EDTA-containing tubes, and after centrifugation at 1500 rpm for 10 min, the plasma was obtained and stored at −20 °C until analysis.

### Pyruvate tolerance test

Due to the prolonged fasting required for this test and the severe damage produced by HFD after six months, there were numerous deaths in the groups of *Ptn*^+/+^-HFD males and females. For this reason, the test was carried out only after three months on HFD or STD. For the test, 16 h-fasted mice were injected intraperitoneally with sodium pyruvate in saline (2 g/kg BW). Blood glucose was measured from the tail vein at the indicated times using a glucometer (Contour, Switzerland).

### Histology and image analysis

Two randomly selected liver sections per animal were deparaffinised, rehydrated and stained with haematoxylin and eosin (H&E) to examine tissue morphology and lipid accumulation. Additional sections were stained with picrosirius red to evaluate collagen and fibrosis. All sections were analysed by optical microscopy (Leica Biosystems). Analysis was performed using *Fiji* software (NIH, Bethesda, USA, Version 1.50f). For H&E staining, five images per mouse (10X objective) were taken to estimate the degree of hepatic steatosis by quantifying the percentage of hepatocytes with lipid accumulation and ballooned hepatocytes. In the groups showing clear steatosis, the pattern of lipid accumulation was studied, distinguishing between macrovesicular and microvesicular accumulation. To study fibrosis, at least 10 blood vessels from each condition were analysed in bright-field for picrosirius red staining (10X objective) to determine perivascular collagen. Type I and type III collagen were determined using polarised light.

### Hepatic lipid analysis

Total lipids were extracted following the Folch method [45], and lipid fractions were separated by thin-layer chromatography in silica gel for quantification as previously described [46].

### Plasma analysis

6 h-fasting plasma glucose and insulin were analysed using an enzymatic colorimetric test (GOD-PAP Roche Diagnostics, Germany) and an immunoassay test (Mercodia, Sweden), respectively. HOMA-IR was calculated using fasting glucose and insulin as previously validated and described by us [47].

Total plasma protein concentration was measured using the Pierce BCA protein method (Thermo-Scientific, Waltham, USA), and major circulating proteins were assessed with the Helena, SAS-MX SP-10 kit. After protein separation by agarose gel electrophoresis in Tris-barbital buffer, gels were stained with Paragon blue 0.5% in 5% acetic acid and scanned for quantification.

Plasma inflammatory markers were assessed using the Olink® Target 96 panels (Uppsala, Sweden), which rely on antibody probes tagged with double oligonucleotide-labelled and microfluidic real-time PCR for quantitative detection. After quality control, data were normalised. Normalised protein expression (NPX) values, an arbitrary unit of log_2_ scale corresponding to higher protein levels, were expressed as fold-change. One control sample that failed quality control was excluded.

Circulating ALT activity was measured using a commercial kit (Spinreact, Spain) according to manufacturer’s instructions.

### Cell culture and treatments: primary hepatocytes

Primary hepatocytes were isolated from non-fasting *Ptn*^+/+^ and *Ptn*^−/−^ mice. After anaesthesia, the suprahepatic vena cava was cannulated through the atrium of the heart and the liver was perfused with two solutions prewarmed at 37 °C. The first solution contained EGTA to wash out blood and eliminate calcium. Then, collagenase solution (10 mg collagenase/mouse; C5138, Sigma-Aldrich) dissociated the ECM, facilitating cell dispersion. The liver was dissected and transferred to a petri dish with hepatocyte attachment medium (AM) (DMEM and Ham´s F-12 medium (1:1) with heat-inactivated 10% fetal bovine serum (FBS), 2 mM glutamine, 15 mM glucose, 20 mM HEPES, 100 U/ml penicillin, 100 µg/ml streptomycin and 1 mM sodium pyruvate), and cells were gently released using 1 mL tips. The suspension was filtered (100 µm-cell strainer) to proceed to purification of hepatocytes by density-based separation. Cells were centrifuged at 50 × g for 5 min at 4 °C. The pellet containing hepatocytes was re-suspended by swirling the tube with 1:1 AM and Percoll solution (GE17-0891–01, Sigma-Aldrich). The suspension was mixed thoroughly and centrifuged for 10 min at 200 × g at RT. Then, the pellet containing live hepatocytes was resuspended in AM and centrifuged 50 × g for 5 min at 4 °C. Finally, hepatocytes were seeded on collagen (C3867-1VL, Sigma-Aldrich) pre-coated plates and maintained in AM for 24 h before treatments. For the different treatments, primary hepatocytes were maintained in serum-free Dulbecco’s Modified Eagle Medium (DMEM) with 5.5 mM glucose. To explore lipid accumulation, cells were treated with 10 nM human recombinant insulin and/or 0.5 µg/mL of human recombinant PTN (R&D Systems) for 48 h.

### Cell culture and treatments: hepatocellular carcinoma Huh7 cell line

The Huh7 cell line was cultured at 37 °C and 5% CO_2_ and grown in DMEM high glucose (4.5 g/L) with L-glutamine (Biowest), 10% (vol./vol.) FBS and 1% (vol./vol.) penicillin/streptomycin (Sigma-Aldrich). Before treatments, Huh7 cells were starved by decreasing FBS to 3%. For analysis of the cellular lipid content, cells were treated with 10 nM recombinant insulin, 0.5 µg/mL of recombinant PTN, or a combination of both for 48 h. The effect of PTN was tested by incubating the cells with 0.5 µg/mL of human recombinant PTN for 0, 0.25, 0.5, 1, 2 or 4 h.

### Intracellular lipid analysis

Oil red (OR) stain was used to evaluate the intracellular lipid content. A stock solution was prepared by dissolving 0.3 g of OR powder (Sigma-Aldrich) in 100 mL of 2-propanol, filtered and stored at RT. The working solution was prepared by diluting 3 parts of stock solution with 2 parts of dH_2_O and filtered before use. The cells were washed twice with PBS and fixed with 4% PFA for 30 min. Next, cells were washed twice with dH_2_O and incubated with 60% 2-propanol for 5 min. After discarding the isopropanol, cells were stained with OR working solution for 15 min at RT and analysed by optical microscopy (Leica Biosystems); the analysis was performed using *Fiji* software (NIH). Images (20X objective) from each well were acquired to estimate the area stained with OR and normalised by cell number.

For the analysis of TAG cell content, cells were washed twice with cold PBS and then homogenised in 100 µL of 5% NP-40/ddH_2_O solution. The samples were heated at 95 °C for 5 min until the NP-40 solution became cloudy and then cooled down to RT. This step was repeated twice to solubilise all TAG. The samples were then frozen at −80 °C for 6 h. TAG were quantified by the glycerol-3-phosphate/peroxidase colorimetric method using a Spinreact kit, in 5 µL of each sample according to the instructions; the absorbance was measured at 505 nm on a SPECTROstarNano microplate reader (BMG Labtech, Germany).

### Protein extraction and immunoblotting

Fifty mg of liver was homogenised in lysis buffer (pH = 7.4; 30 mM HEPES, 5 mM EDTA, 1% Triton X-100, 0.5% sodium deoxycholate, 8 mM Na_3_VO_4_, 1 mM NaF, and 2 mM of the protease inhibitor Pefabloc (Roche Diagnostics)). An equal amount of protein (measured using the Pierce BCA protein method) was subjected to SDS-PAGE, transferred to PVDF membranes (Amersham-GE Healthcare, UK), and incubated with the antibodies indicated in Table S1. Vinculin was used as a loading control. After the incubation with the corresponding horseradish peroxidase-conjugated antibody, proteins were visualised by enhanced chemiluminescence (ECL) (Amersham; #RPN2236; UK) using the ChemiDoc Imaging system (Bio-Rad, USA) and quantified by densitometry using Image Lab image acquisition and analysis software (Bio-Rad).

### Quantitative real-time PCR (qPCR)

Total RNA from liver and cells was isolated using the Total RNA Isolation Kit (Nzytech, Portugal). First-strand cDNA was synthesised using the first-strand cDNA Synthesis Kit (Nzytech), and 5 μg of RNA was reverse-transcribed to DNA. qPCR analysis was performed using the SYBR green method (Quantimix Easy kit, Biotools, Spain) in a CFX96 Real-Time System (Bio-Rad). The relative expression of each gene was normalised in liver samples using actin (*Actb*) and ribosomal protein L13 (*Rpl13*) as housekeeping genes and in cells with glyceraldehyde-3-phosphate dehydrogenase (*GAPDH*) and ribosomal protein L30 (*RPL30*). The data were analysed by the Livak method. The primer sequences are shown in Table S2.

### Statistical analysis

Statistical analyses were performed using IBM-SPSS v.25 software. Data are shown as mean and the standard error. Normality was assessed by the Shapiro–Wilk test, and parametric tests were used when data met normality. For the in vivo studies with 3 variables (sex, genotype, and diet), a three-way ANOVA test was applied. When relevant, a two-way ANOVA was used to better dissect the effect of each variable, excluding the non-significant variable when the three-way ANOVA results allowed. When appropriate, a repeated-measures ANOVA was performed. When only two experimental groups were compared, an unpaired T-test was used. For the in vitro studies, to evaluate the response to insulin or PTN, two-way or one-way ANOVA was applied as appropriate. When data did not meet normality, a Kruskal–Wallis test was used. Bonferroni’s *post-hoc* analysis was used to compare differences between groups when an interaction between variables was found. Statistical significance refers to the individual factors and their interaction from the corresponding test. In the figures of the in vivo studies, significant differences are represented with (*) for differences between diets, (#) genotypes, and (&) sexes. For the figures of the in vitro studies, significant differences are represented with (*) or (#) as indicated in the figure legends. A 95% confidence interval was used for all statistical comparisons. The complete statistical analysis is shown in Table S3.

## Supplementary Information

Supplementary Material 1.

## Data Availability

The datasets used and/or analysed during the current study are available from the corresponding author upon reasonable request.
