# Supplementary material for: Deletion of Pleiotrophin protects against high-fat diet-induced liver metabolic disease, independently of the sexual dimorphism in dietary response
Source: Mol Biomed. 2026 Aug 20;7:146. doi: 10.1186/s43556-026-00547-9 (PMC13493653; doi:10.1186/s43556-026-00547-9)
Supplement: Supplementary file 1 [file 43556_2026_547_MOESM1_ESM.pdf]

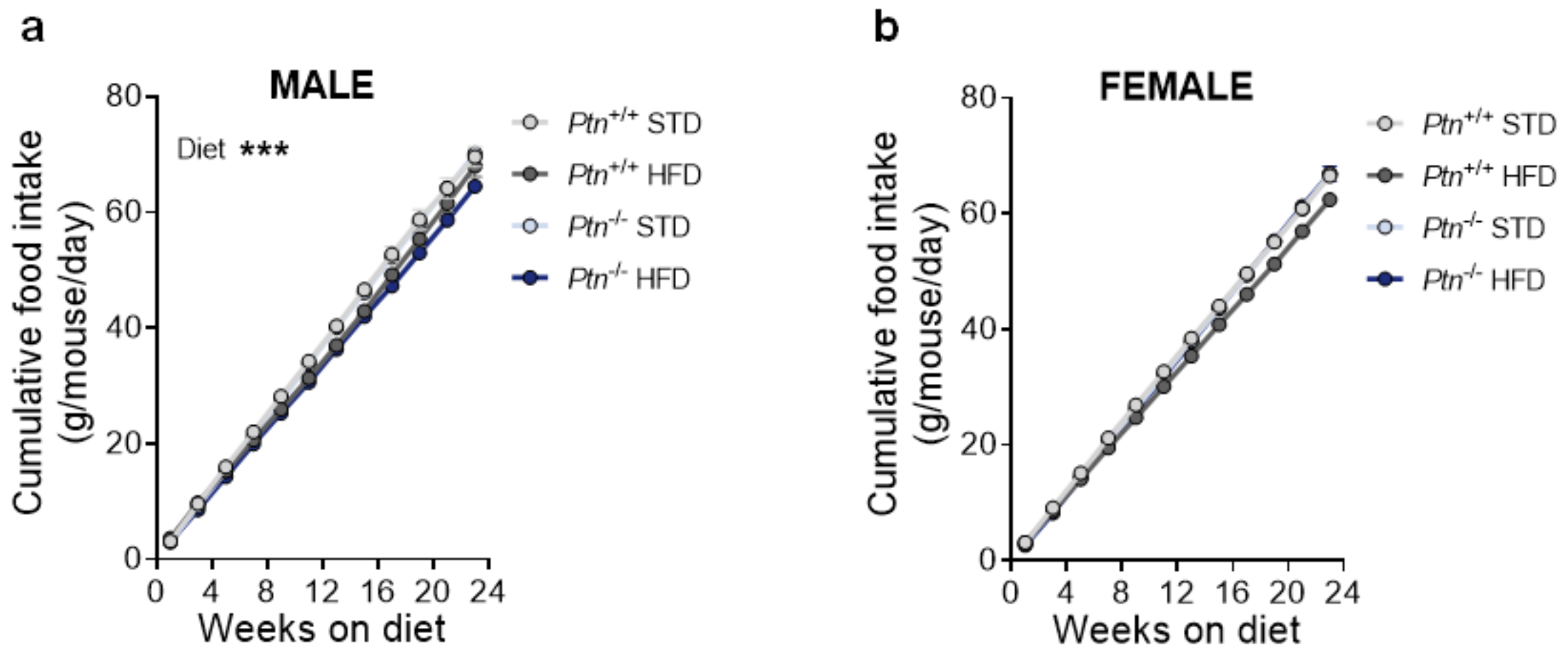

Fig. S1. High-fat diet feeding and *Ptn* deletion effects on food intake. Cumulative food intake over time in male (a) and female (b) $Ptn^{+/+}$ and $Ptn^{-/-}$ mice fed with a STD (18% kcal fat) or HFD (60% kcal fat) for 6 months starting at 3 months of age. Data are presented as mean ± SEM (n = 7 animals per group). Differences between diets are represented with: ***$p < 0.001$.

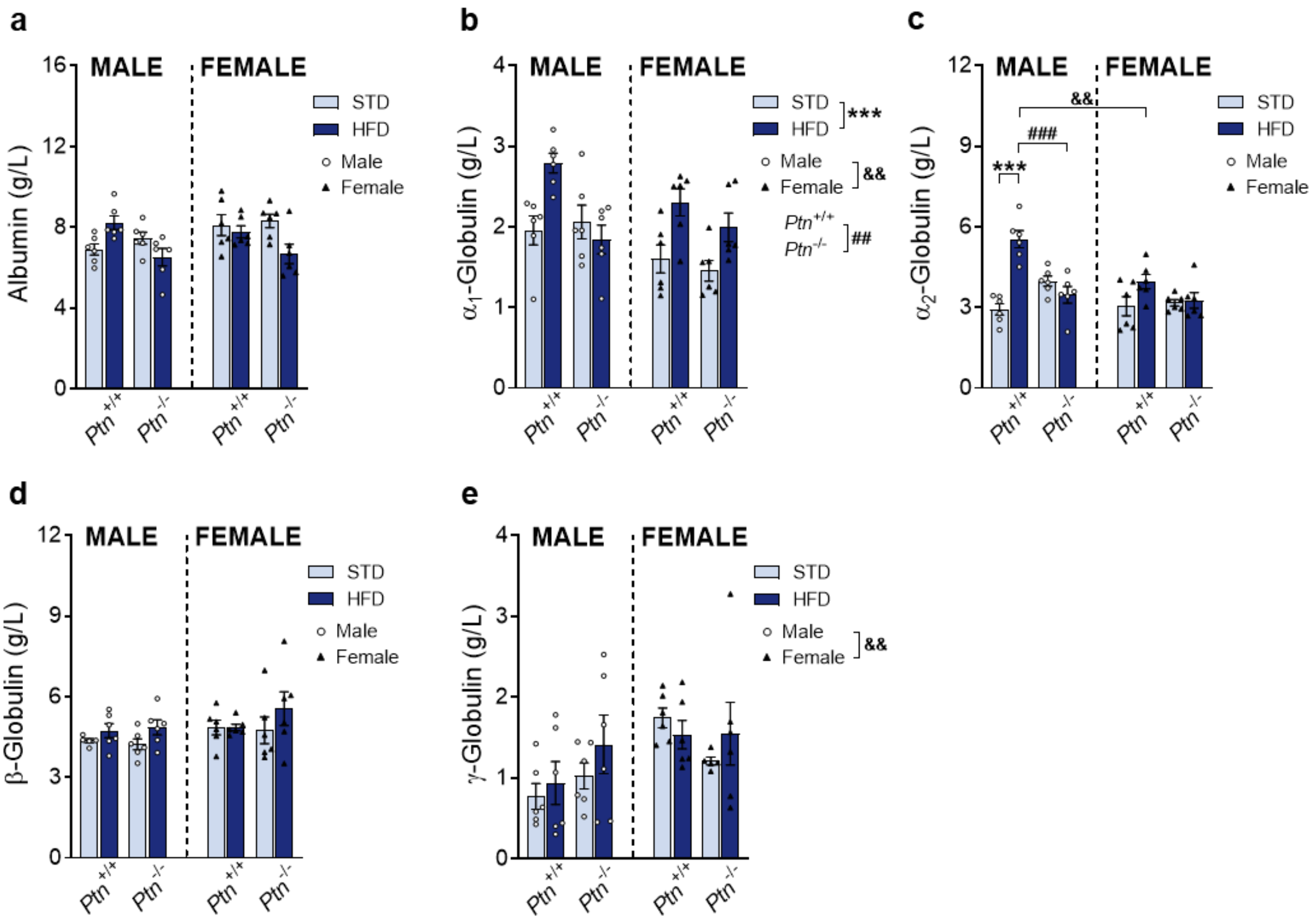

Fig. S2. High-fat diet feeding and *Ptn* deletion effects on serum proteins. Male and female *Ptn*$^{+/+}$ and *Ptn*$^{-/-}$ mice were fed a STD (18% kcal fat) or a HFD (60% kcal fat) for 6 months starting at 3 months of age. Albumin (a), α_1-globulin (b), α_2-globulin (c), β-globulin (d), and γ-globulin (e) levels quantified from electrophoretic profiles. Data are presented as mean ± SEM (n= 6 animals per group). Differences between diets are represented with: $^{***}p < 0.001$; between genotypes with: $^{\#\#}p < 0.01$; $^{\#\#\#}p < 0.001$; and between sexes with: $^{\&\&}p < 0.01$.

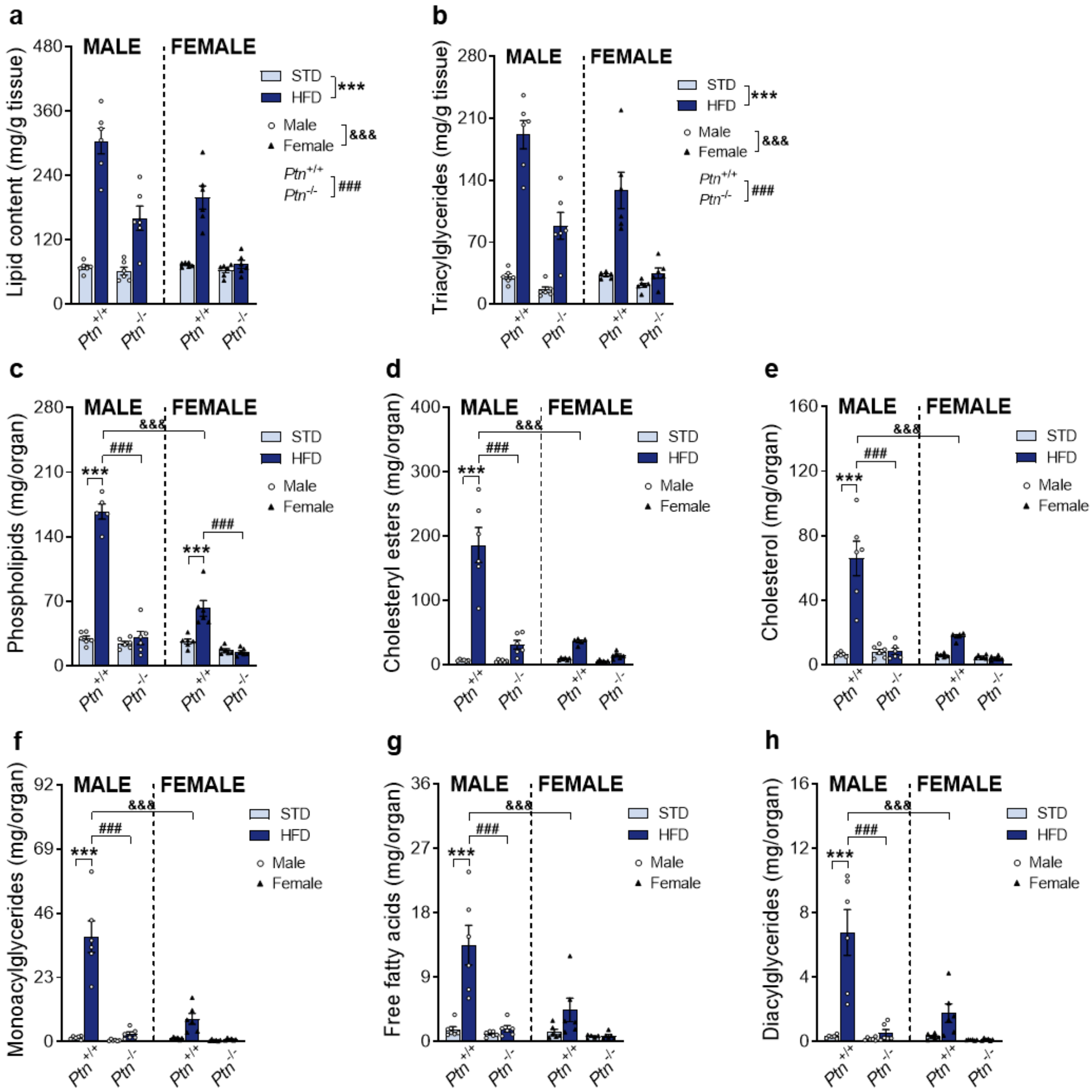

Fig. S3. High-fat diet feeding and *Ptn* deletion effects on hepatic lipid content. Male and female $Ptn^{+/+}$ and $Ptn^{-/-}$ mice were fed a STD (18% kcal fat) or a HFD (60% kcal fat) for 6 months starting at 3 months of age. Hepatic lipid content (a) and triacylglycerides (b) (mg/g tissue) determined by the Folch extraction method. Phospholipids (c), cholesteryl esters (d), cholesterol (e), monoacylglycerides (f), free fatty acids (g), and diacylglycerides (h) content in liver lipid extracts. Data are presented as mean ± SEM (n = 7 animals per group; circles correspond to males and triangles correspond to females). Differences between diets are represented with: $^{***}p < 0.001$; between genotypes with: $^{\#\#\#}p < 0.001$; and between sexes with: $^{\&\&\&}p < 0.001$.

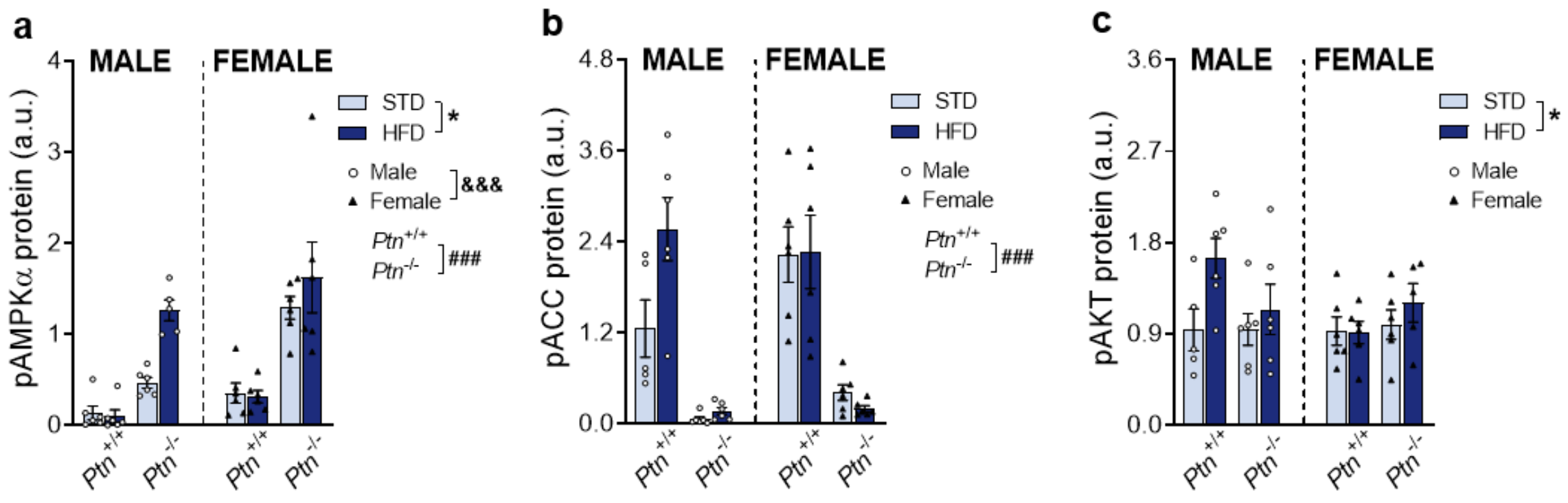

Fig. S4. Hepatic protein levels of phospho-AMP-activated protein kinase α (AMPKα) (Thr172) (a), phospho-acetyl-CoA carboxylase (ACC) (Ser79) (b), phospho-protein kinase B (AKT) (Ser472) (c) were determined by Western blot analysis. Vinculin was used as a loading control. Liver samples from 9-month-old male and female *Ptn*$^{+/+}$ and *Ptn*$^{-/-}$ mice were collected after 6 months of feeding with either STD (18% kcal fat) or HFD (60% kcal fat) starting at 3 months of age. Data are presented as mean ± SEM (n = 6 animals per group; circles correspond to males and triangles correspond to females). Differences between diets are represented with: *$p < 0.05$; between genotypes with: $^{\#\#\#}p < 0.001$; and between sexes with: $^{\&\&\&}p < 0.001$.

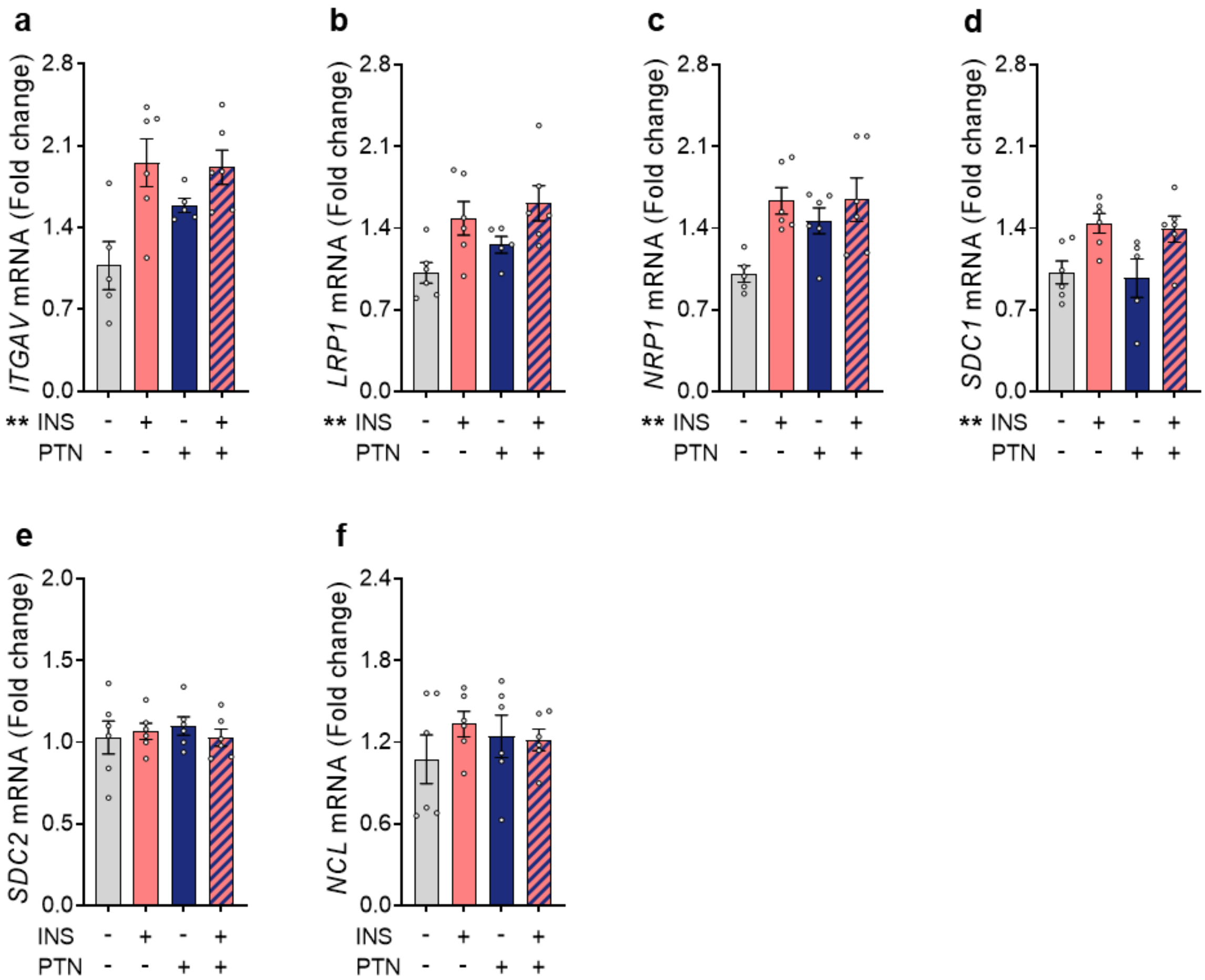

Fig. S5. mRNA of PTN receptors in the Huh7 cell line. Huh-7 cells were cultured in high-glucose DMEM supplemented with 10% fetal bovine serum and 1% penicillin/streptomycin. For treatments, cells were serum-starved (3% FBS) and incubated for 48 h with 10 nM human recombinant insulin, 0.5 µg/mL recombinant human PTN, or both. mRNA levels of integrin subunit alpha V (*ITGAV*) (a), LDL receptor-related protein 1 (*LRP1*) (b), neuropilin 1 (*NRP1*) (c), syndecan 1 (*SDC1*) (d), syndecan 2 (*SDC2*) (e), and nucleolin (*NCL*) (f) were determined by quantitative real-time PCR (qPCR) and normalized to *GAPDH* and *RPL30* expression. mRNA expression of anaplastic lymphoma kinase (*ALK*) and protein tyrosine phosphatase receptor type Z1 (*PTPRZ1*) was below detection limits. Data are presented as mean ± SEM (n = 6 per group). The treatment effects are represented with: $^{**}p < 0.01$.

Table S1. Primary antibodies used for western blot analysis.

Primary Ab	Reference
AQP9	Invitrogen; #PA5-76416; 1:500; rabbit
DGAT2	Thermo Fisher; #PA5-103785; 1:1000; rabbit
AMPKα	Cell Signaling; #2532; 1:500; rabbit
pAMPKα (Thr172)	Cell Signaling; #2531; 1:1000; rabbit
ACC	Millipore; #MABS2024; 1:500; mouse
pACC (Ser79)	Millipore; #07-303; 1:500; rabbit
AKT	Cell Signaling; #CS2920; 1:1000; mouse
pAKT (Ser473)	Cell Signaling; #CS4060; 1:1000; rabbit
Vinculin	Sigma-Aldrich; #V9264; 1:10000; mouse

AQP9: anti-aquaporin 9; GDAT2: anti-diacylglycerol O-acyltransferase 2; AMPKα: anti-AMP-activated protein kinase α; pAMPKα: anti-phospho-AMPKα (Thr172); ACC: anti-acetyl-CoA carboxylase; pACC: anti-phospho-ACC (Ser79); AKT: anti-protein kinase B; pAKT: anti-phospho-protein kinase B (Ser473), and vinculin.

Table S2. Primer sets used for qPCR analysis.

Gene	Primer Forward	Primer Reverse
Actb	5'-TGTTACCAACTGGGACGACA-3'	5'-GGGGTGTTGAAGGTCTCAAA-3'
Itga1	5’-CCTTCCCTCGGATGTGAGTCA-3’	5’-AAGTTCTCCCCGTATGGTAAGA-3’
Itgb3	5’-CCACACGAGGCGTGAACTC-3’	5’-CTTCAGGTTACATCGGGGTGA-3’
Lrp1	5’-CTCCTGGACGTGTGATCTGG-3’	5’-TGGCGTGTGTCTCATCACTG-3’
Mdk	5'-TGATGGGAGCACTGGCAC-3'	5'-CATTGTACCGCGCCTTCTT-3'
Ncl	5’-CATGGTGAAGCTCGCAAAG-3’	5’-CATCTTCACTATCCTCTTCCACCT-3’
Nrp1	5’-GGAGCTACTGGGCTGTGAAG-3’	5’-ACCGTATGTCGGGAACTCTG-3’
Ptn	5'-TTGGGGAGAATGTGACCTCAATAC-3'	5'-GGCTTGGAGATGGTGACAGTTTTC-3'
Rpl13	5'-GGTGCCCTACAGTTAGATACCAC-3'	5'-TTTGTTTCGCCTCCTTGGGTC-3'
Sdc1	5'-TTCTTGCGATACACTCTGGTGC-3'	5'-CGGGATTGAATGTTCTTGTCGT-3'
Sdc3	5'-TGATGATGAACTAGACGACC-3'	5'-AGAAAGGAGTTCCTCAAATGG-3'

For tissue analysis. *Actb*: Actin beta; *Itga1*: Integrin alpha 1; *Itgb3*: Integrin beta 3; *Lrp1*: Low density lipoprotein receptor-related protein 1; *Mdk*: Midkine; *Ncl*: Nucleolin; *Nrp1*: Neuropilin 1; *Ptn*: Pleiotrophin; *Rpl13*: Ribosomal protein L13; *Sdc1*: Syndecan 1; *Sdc3*: Syndecan 3.

Gene	Primer Forward	Primer Reverse
ALK	5’-CTGTCTCATCGCAGCCGATA-3’	5’-TGGGCATCTCCTTAGAACGC-3’
GAPDH	5’-TGCACCACCAACTGCTTAGC-3’	5’-GGCATGGACTGTGGTCATGAG-3’
ITGAV	5’-CTGCCTGGAACAGCTCTCAA-3’	5’-CTTGGAGTGACTTGGGGACC-3’
LRP1	5’-CTCCTGGACGTGTGATCTGG-3’	5’-TGGCGTGTGTCTCATCACTG-3’
NCL	5’-AAGGCACAGAACCGACTACG-3’	5’-CCAACGCTTTCTCCAGGTCT-3’
NRP1	5’-CGCTACGACCGGCTAGAAAT-3’	5’-GGAGAGAATGCCCGATGAGG-3’
PTPRZ1	5’-GCCTGGATTGGGCTAATGGA-3’	5’-TGAAACTCCTCCGCTGACAC-3’
RPL30	5’-AAAGCTGGGCAGTTGTTAGC-3’	5’-AAGACGAAAAAGTCGCTGGA-3’
SDC1	5’-CTGGGCTGGAATCAGGAATA -3’	5’-ACACACAGCACAGAGGCAAG-3’
SDC2	5’-GAGCTGACAACATCTCGACCA-3’	5’-CTCTTCGGCTGGGTCCATTT-3’

For cell analysis. *ALK*: ALK receptor tyrosine kinase; *GAPDH*: glyceraldehyde-3-phosphate dehydrogenase; *ITGAV*: integrin subunit alpha V; *LRP1*: Low density lipoprotein receptor-related protein 1; *NCL*: Nucleolin; *NRP1*: Neuropilin 1; *PTPRZ1*: Protein tyrosine phosphatase receptor type Z1; *RLP30*: ribosomal protein L30; *SDC1*: Syndecan 1; *SDC2*: Syndecan 2.

Table S3. Complete statistical analysis.

Statistical analysis from Fig. 1.

Test	Genotype	Diet	Interaction	
Two-way repeated measures ANOVA (Fig. 1a)	$F_{1, 24} = 75.961$; $p < 0.001$	$F_{1, 24} = 193.314$; $p < 0.001$	$F_{1, 24} = 58.525$; $p < 0.001$	
Two-way repeated measures ANOVA (Fig. 1b)	$F_{1, 24} = 37.078$; $p < 0.001$	$F_{1, 24} = 105.375$; $p < 0.001$	$F_{1, 24} = 40.741$; $p < 0.001$	
	**Genotype**	**Diet**	**Sex**	**Interaction**
Three-way ANOVA (Fig. 1c)	$F_{1, 55} = 108.697$; $p < 0.001$	$F_{1, 55} = 129.295$; $p < 0.001$	$F_{1, 55} = 32.333$; $p < 0.001$	n.s.
Three-way ANOVA (Fig. 1e)	$F_{1, 55} = 29.140$; $p < 0.001$	$F_{1, 55} = 4.433$; $p = 0.041$	n.s.	n.s.
Three-way ANOVA (Fig. 1f)	$F_{1, 55} = 35.746$; $p < 0.001$	$F_{1, 55} = 32.018$; $p < 0.001$	$F_{1, 55} = 22.569$; $p < 0.001$	$F_{1, 55} = 7.292$; $p = 0.01$
Three-way ANOVA (Fig. 1g)	$F_{1, 55} = 39.678$; $p < 0.001$	$F_{1, 55} = 34.101$; $p < 0.001$	$F_{1, 55} = 22.291$; $p < 0.001$	$F_{1, 55} = 9.266$; $p = 0.004$
Three-way ANOVA (Fig. 1h)	$F_{1, 53} = 133.666$; $p < 0.001$	$F_{1, 53} = 78.995$; $p < 0.001$	$F_{1, 53} = 13.677$; $p = 0.001$	n.s.
	**Genotype**	**Diet**	**Interaction**	
Two-way repeated measures ANOVA (Fig. 1i)	$F_{1, 24} = 107.414$; $p < 0.001$	$F_{1, 24} = 54.787$; $p < 0.001$	$F_{1, 24} = 25.200$; $p < 0.001$	
Two-way repeated measures ANOVA (Fig. 1j)	$F_{1, 23} = 104.806$; $p < 0.001$	$F_{1, 23} = 24.080$; $p < 0.001$	$F_{1, 23} = 21.410$; $p < 0.001$	
	**Genotype**	**Diet**	**Sex**	**Interaction**
Three-way ANOVA (Fig. 1k)	$F_{1, 53} = 236.007$; $p < 0.001$	$F_{1, 53} = 76.982$; $p < 0.001$	$F_{1, 53} = 12.083$; $p = 0.001$	n.s.

Statistical analysis from Fig. 2.

Test	Diet	Sex	Interaction	
Two-way ANOVA (Fig. 2a)	n.s.	n.s.	$F_{1, 26} = 10.278$; $p = 0.004$	
	**Genotype**	**Diet**	**Sex**	**Interaction**
Three-way ANOVA (Fig. 2b)	$F_{1, 55} = 6.635$; $p = 0.013$	n.s.	$F_{1, 55} = 54.570$; $p < 0.001$	$F_{1, 55} = 6.878$; $p = 0.012$
Three-way ANOVA (Fig. 2c)	$F_{1, 54} = 100.751$; $p < 0.001$	$F_{1, 54} = 8.190$; $p = 0.006$	$F_{1, 54} = 19.556$; $p < 0.001$	n.s.
Three-way ANOVA (Fig. 2d)	n.s.	$F_{1, 55} = 6.638$; $p = 0.013$	$F_{1, 55} = 14.312$; $p < 0.001$	n.s.
Three-way ANOVA (Fig. 2e)	F1, 53 = 3.957; p = 0.053	F1, 53 = 6.497; p = 0.014	F1, 53 = 12.913; p = 0.001	n.s.
Three-way ANOVA (Fig. 2f)	n.s.	n.s.	n.s.	n.s.
	**Genotype**	**Diet**	**Interaction**	
Two-way ANOVA (Fig. 2f')	n.s.	n.s.	$F_{1, 53} = 34.822$; $p < 0.001$	

Statistical analysis from Fig. 3.

Test	Genotype	Diet	Sex	Interaction
Three-way ANOVA (Fig. 3a)	$F_{1, 48} = 43.387$; $p < 0.001$	$F_{1, 48} = 9.134$; $p = 0.004$	n.s.	n.s.
	**Genotype**	**Diet**	**Interaction**	
Two-way ANOVA (Fig. 3a')	$F_{1, 48} = 47.129$; $p < 0.001$	$F_{1, 48} = 9.565$; $p = 0.003$	$F_{1, 48} = 14.251$; $p < 0.001$	
	**Genotype**	**Diet**	**Sex**	**Interaction**
Three-way ANOVA (Fig. 3b)	n.s.	$F_{1, 47} = 5.179$; $p = 0.028$	n.s.	$F_{1, 47} = 4.555$; $p = 0.039$
	**Genotype**	**Diet**	**Sex**	
Kruskal-Wallis; Pre-Albumin (Fig. 3c.1)	H(1) = 18.161; $p < 0.001$	H(1) = 11.585; $p = 0.001$	n.s.	
	**Genotype**	**Diet**	**Sex**	**Interaction**
Three-way ANOVA; Albumin (Fig. 3c.2)	n.s.	$F_{1, 46} = 21.097$; $p < 0.001$	n.s.	n.s.
Three-way ANOVA; α_1-Globulin (Fig. 3c.3)	$F_{1, 47} = 5.259$; $p = 0.027$	$F_{1, 47} = 9.674$; $p = 0.003$	$F_{1, 47} = 13.226$; $p = 0.001$	n.s.
Three-way ANOVA; α_2-Globulin (Fig. 3c.4)	n.s.	$F_{1, 46} = 23.657$; $p < 0.001$	$F_{1, 46} = 41.660$; $p < 0.001$	$F_{1, 46} = 4.972$; $p = 0.032$
Three-way ANOVA; β-Globulin (Fig. 3c.5)	n.s.	n.s.	n.s.	n.s.
Three-way ANOVA; γ-Globulin (Fig. 3c.6)	n.s.	n.s.	$F_{1, 46} = 8.305$; $p = 0.006$	n.s.
	**Genotype**	**Diet**	**Sex**	
Kruskal-Wallis (Fig. 3d)	H(1) = 18.161; $p < 0.001$	H(1) = 12.157; $p < 0.001$	n.s.	
	**Genotype**	**Diet**	**Sex**	**Interaction**
Three-way ANOVA (Fig. 3e)	$F_{1, 47} = 7.138$; $p = 0.011$	$F_{1, 47} = 21.144$; $p = 0.001$	$F_{1, 47} = 12.104$; $p = 0.01$	$F_{1, 47} = 8.606$; $p = 0.006$
Three-way ANOVA (Fig. 3f)	$F_{1, 55} = 9.920$; $p = 0.003$	$F_{1, 55} = 20.955$; $p < 0.001$	n.s.	n.s.
	**Genotype**	**Diet**	**Interaction**	
Two-way ANOVA (Fig. 3f')	$F_{1, 55} = 10.417$; $p = 0.002$	$F_{1, 55} = 22.005$; $p < 0.001$	$F_{1, 55} = 17.983$; $p < 0.001$	
	**Genotype**	**Diet**	**Sex**	**Interaction**
Three-way ANOVA (Fig. 3g)	$F_{1, 55} = 143.726$; $p < 0.001$	$F_{1, 55} = 23.365$; $p < 0.001$	n.s.	n.s.
	**Genotype**	**Diet**	**Interaction**	
Two-way ANOVA (Fig. 3g')	$F_{1, 55} = 140.886$; $p < 0.001$	$F_{1, 55} = 22.903$; $p < 0.001$	$F_{1, 55} = 16.961$; $p < 0.001$	

Statistical analysis from Fig. 4.

Test	Genotype	Diet	Sex	Interaction
Three-way ANOVA (Fig. 4a)	$F_{1, 55} = 376.119$; $p < 0.001$	$F_{1, 55} = 221.875$; $p < 0.001$	$F_{1, 55} = 115.149$; $p < 0.001$	$F_{1, 55} = 74.624$; $p < 0.001$
Three-way ANOVA (Fig. 4b)	$F_{1, 54} = 260.181$; $p < 0.001$	$F_{1, 54} = 36.032$; $p < 0.001$	$F_{1, 54} = 51.618$; $p < 0.001$	$F_{1, 54} = 75.264$; $p < 0.001$
Three-way ANOVA (Fig. 4d)	$F_{1, 53} = 2319.832$; $p < 0.001$	$F_{1, 53} = 2990.280$; $p < 0.001$	$F_{1, 53} = 219.541$; $p < 0.001$	$F_{1, 53} = 155.799$; $p < 0.001$
	**Genotype**	**Diet**	**Sex**	
Kruskal-Wallis (Fig. 4e)	H(1) = 15.626; $p < 0.001$	H(1) = 15.549; $p < 0.001$	n.s.	
	**Sex**			
T-Student (Fig. 4f)	$t_{13} = 5.029$; $p < 0.001$			
	**Genotype**	**Diet**	**Sex**	**Interaction**
Three-way ANOVA (Fig. 4g)	$F_{1, 47} = 103.502$; $p < 0.001$	$F_{1, 47} = 123.361$; $p < 0.001$	$F_{1, 47} = 48.587$; $p < 0.001$	$F_{1, 47} = 30.877$; $p < 0.001$
Three-way ANOVA; Cholesteryl esters (Fig. 4h.1)	$F_{1, 46} = 32.502$; $p < 0.001$	$F_{1, 46} = 69.387$; $p < 0.001$	n.s.	$F_{1, 46} = 7.804$; $p = 0.008$
Three-way ANOVA; Cholesterol (Fig. 4h.2)	$F_{1, 47} = 22.165$; $p < 0.001$	$F_{1, 47} = 53.043$; $p < 0.001$	n.s.	n.s.
Three-way ANOVA; Triacylglycerides (Fig. 4h.3)	$F_{1, 45} = 59.750$; $p < 0.001$	$F_{1, 45} = 139.361$; $p < 0.001$	n.s.	$F_{1, 45} = 4.175$; $p = 0.048$
Three-way ANOVA; Free fatty acids (Fig. 4h.4)	n.s.	$F_{1, 45} = 4.972$; $p = 0.032$	n.s.	n.s.
Three-way ANOVA; Diacylglycerides (Fig. 4h.5)	$F_{1, 45} = 10.618$; $p = 0.002$	n.s.	n.s.	n.s.
Three-way ANOVA; Monoacylglycerides (Fig. 4h.6)	$F_{1, 46} = 25.626$; $p < 0.001$	$F_{1, 46} = 16.448$; $p < 0.001$	n.s.	n.s.
Three-way ANOVA; Phospholipids (Fig. 4h.7)	$F_{1, 47} = 22.988$; $p < 0.001$	$F_{1, 47} = 169.335$; $p < 0.001$	n.s.	n.s.
	**Genotype**	**Diet**	**Interaction**	
Two-way ANOVA; Cholesterol (Fig. 4h.2')	$F_{1, 47} = 21.499$; $p < 0.001$	$F_{1, 47} = 51.218$; $p < 0.001$	$F_{1, 47} = 12.279$; $p = 0.001$	
Two-way ANOVA; Free fatty acids (Fig. 4h.4')	n.s.	$F_{1, 45} = 5.680$; $p = 0.022$	n.s.	
Two-way ANOVA; Diacylglycerides (Fig. 4h.5')	$F_{1, 45} = 11.303$; $p = 0.002$	n.s.	n.s.	
Two-way ANOVA; Monoacylglycerides (Fig. 4h.6')	$F_{1, 46} = 24.066$; $p < 0.001$	$F_{1, 46} = 14.630$; $p < 0.001$	n.s.	
Two-way ANOVA; Phospholipids (Fig. 4h.7')	$F_{1, 47} = 21.050$; $p < 0.001$	$F_{1, 47} = 155.055$; $p < 0.001$	n.s.	
	**Genotype**	**Diet**	**Sex**	**Interaction**
Three-way ANOVA (Fig. 4i)	$F_{1, 47} = 104.262$; $p < 0.001$	$F_{1, 47} = 126.893$; $p < 0.001$	$F_{1, 47} = 43.558$; $p < 0.001$	$F_{1, 47} = 27.712$; $p < 0.001$

Statistical analysis from Fig. 5.

**Test**	**Genotype**	**Diet**	**Sex**	**Interaction**
Three-way ANOVA (Fig. 5a)	n.s.	$F_{1, 46} = 17.287$; $p < 0.001$	n.s.	n.s.
	**Genotype**	**Diet**	**Interaction**	
Two-way ANOVA (Fig. 5a')	n.s.	$F_{1, 46} = 16.037$; $p < 0.001$	$F_{1, 46} = 4.685$; $p = 0.036$	
	**Genotype**	**Diet**	**Sex**	**Interaction**
Three-way ANOVA (Fig. 5b)	$F_{1, 46} = 9.623$; $p = 0.004$	$F_{1, 46} = 3.053$; $p = 0.088$	n.s.	n.s.
	**Genotype**	**Diet**	**Interaction**	
Two-way ANOVA (Fig. 5b')	$F_{1, 46} = 9.650$; $p = 0.003$	$F_{1, 46} = 3.107$; $p = 0.085$	$F_{1, 46} = 14.950$; $p < 0.001$	
	**Genotype**	**Diet**	**Sex**	**Interaction**
Three-way ANOVA (Fig. 5c)	$F_{1, 46} = 52.619$; $p < 0.001$	n.s.	$F_{1, 46} = 17.014$; $p < 0.001$	n.s.
Three-way ANOVA (Fig. 5d)	$F_{1, 45} = 46.641$; $p < 0.001$	n.s.	n.s.	n.s.
Three-way ANOVA (Fig. 5e)	$F_{1, 46} = 103.876$; $p < 0.001$	$F_{1, 46} = 8.846$; $p = 0.005$	$F_{1, 46} = 22.149$; $p < 0.001$	n.s.
Three-way ANOVA (Fig. 5f)	$F_{1, 42} = 39.783$; $p < 0.001$	$F_{1, 42} = 16.111$; $p < 0.001$	n.s.	n.s.
Three-way ANOVA (Fig. 5g)	$F_{1, 46} = 12.981$; $p < 0.001$	n.s.	$F_{1, 46} = 11.656$; $p = 0.002$	n.s.
Three-way ANOVA (Fig. 5h)	$F_{1, 46} = 6.231$; $p = 0.017$	$F_{1, 46} = 6.993$; $p = 0.001$	$F_{1, 46} = 12.520$; $p = 0.001$	$F_{1, 46} = 9.523$; $p = 0.004$

Statistical analysis from Fig. 6.

**Test**	**Genotype**	**Diet**	**Sex**	**Interaction**
Three-way ANOVA (Fig. 6b)	$F_{1, 55} = 159.488$; $p < 0.001$	$F_{1, 55} = 75.980$; $p < 0.001$	$F_{1, 55} = 56.668$; $p < 0.001$	$F_{1, 55} = 61.836$; $p < 0.001$
Three-way ANOVA (Fig. 6c)	$F_{1, 55} = 292.150$; $p < 0.001$	$F_{1, 55} = 99.870$; $p < 0.001$	$F_{1, 55} = 101.040$; $p < 0.001$	$F_{1, 55} = 106.376$; $p < 0.001$
Three-way ANOVA (Fig. 6d)	$F_{1, 55} = 256.394$; $p < 0.001$	$F_{1, 55} = 86.523$; $p < 0.001$	$F_{1, 55} = 66.864$; $p < 0.001$	$F_{1, 55} = 106.413$; $p < 0.001$
Three-way ANOVA; Type I collagen ratio (Fig. 6e.1)	$F_{1, 55} = 7.118$; $p = 0.01$	$F_{1, 55} = 7.732$; $p = 0.008$	n.s.	n.s.
Three-way ANOVA; Type III collagen ratio (Fig. 6e.2)	$F_{1, 55} = 22.709$; $p < 0.001$	$F_{1, 55} = 18.079$; $p < 0.001$	n.s.	$F_{1, 55} = 4.536$; $p = 0.038$

Statistical analysis from Fig. 7.

Test	Insulin	PTN	Interaction
Two-way ANOVA (Fig. 7b)	$F_{1, 23} = 37.667$; $p < 0.001$	$F_{1, 23} = 27.515$; $p < 0.001$	$F_{1, 23} = 26.498$; $p < 0.001$
Two-way ANOVA (Fig. 7c)	n.s.	$F_{1, 23} = 103.786$; $p < 0.001$	n.s.
Two-way ANOVA (Fig. 7d)	n.s.	n.s.	n.s.
Two-way ANOVA (Fig. 7e)	n.s.	$F_{1, 20} = 15.540$; $p = 0.001$	n.s.
	**Genotype**		
Unpared T-Student (Fig. 7f)	t(10) = 10,473; $p < 0.001$		
Unpared T-Student (Fig. 7g)	t(6,625) = 3,995; $p = 0.006$		
	**Insulin**	**PTN**	**Interaction**
Two-way ANOVA (Fig. 7i)	$F_{1, 26} = 260.053$; $p < 0.001$	$F_{1, 26} = 277.357$; $p < 0.001$	$F_{1, 26} = 95.581$; $p < 0.001$
Two-way ANOVA (Fig. 7j)	$F_{1, 23} = 10.878$; $p = 0.004$	$F_{1, 23} = 9.994$; $p = 0.005$	n.s.
	**PTN**		
One-way ANOVA (Fig. 7k)	$F_{5, 28} = 2.789$; $p = 0.041$		
One-way ANOVA (Fig. 7l)	$F_{5, 28} = 10.627$; $p < 0.001$		

Statistical analysis from Fig. S1.

Test	Genotype	Diet	Interaction
Two-way repeated measures ANOVA (Fig. S1a)	n.s.	$F_{1, 21} = 14.218$; $p = 0.001$	n.s.
Two-way repeated measures ANOVA (Fig. S1b)	n.s.	$F_{1, 24} = 3.912$; $p = 0.06$	n.s.

Statistical analysis from Fig. S2.

Test	Genotype	Diet	Sex	Interaction
Three-way ANOVA (Fig. S2a)	n.s.	n.s.	n.s.	n.s.
Three-way ANOVA (Fig. S2b)	$F_{1, 47} = 7.357$; $p = 0.01$	$F_{1, 47} = 15.061$; $p < 0.001$	$F_{1, 47} = 7.329$; $p = 0.01$	n.s.
Three-way ANOVA (Fig. S2c)	$F_{1, 47} = 4.383$; $p = 0.043$	$F_{1, 47} = 16.813$; $p < 0.001$	$F_{1, 47} = 10.543$; $p = 0.002$	$F_{1, 47} = 9.058$; $p = 0.005$
Three-way ANOVA (Fig. S2d)	n.s.	n.s.	n.s.	n.s.
Three-way ANOVA (Fig. S2e)	n.s.	n.s.	$F_{1, 46} = 7.524$; $p = 0.009$	n.s.

Statistical analysis from Fig. S3.

Test	Genotype	Diet	Sex	Interaction
Three-way ANOVA (Fig. S3a)	$F_{1, 47}$ = 47.878; p < 0.001	$F_{1, 47}$ = 129.742; p < 0.001	$F_{1, 47}$ = 20.133; p < 0.001	n.s.
Three-way ANOVA (Fig. S3b)	$F_{1, 47}$ = 51.152; p < 0.001	$F_{1, 47}$ = 120.519; p < 0.001	$F_{1, 47}$ = 12.410; p = 0.001	n.s.
Three-way ANOVA (Fig. S3c)	$F_{1, 46}$ = 197.142; p < 0.001	$F_{1, 46}$ = 156.803; p < 0.001	$F_{1, 46}$ = 85.623; p < 0.001	$F_{1, 46}$ = 43.741; p < 0.001
Three-way ANOVA (Fig. S3d)	$F_{1, 46}$ = 39.940; p < 0.001	$F_{1, 46}$ = 69.582; p < 0.001	$F_{1, 46}$ = 32.621; p < 0.001	$F_{1, 46}$ = 22.144; p < 0.001
Three-way ANOVA (Fig. S3e)	$F_{1, 46}$ = 38.455; p < 0.001	$F_{1, 46}$ = 39.102; p < 0.001	$F_{1, 46}$ = 24.681; p < 0.001	$F_{1, 46}$ = 19.959; p < 0.001
Three-way ANOVA (Fig. S3f)	$F_{1, 47}$ = 53.183; p < 0.001	$F_{1, 47}$ = 57.593; p < 0.001	$F_{1, 47}$ = 27.589; p < 0.001	$F_{1, 47}$ = 20.581; p < 0.001
Three-way ANOVA (Fig. S3g)	$F_{1, 46}$ = 23.600; p < 0.001	$F_{1, 46}$ = 21.223; p < 0.001	$F_{1, 46}$ = 9.960; p = 0.003	$F_{1, 46}$ = 5.653; p = 0.022
Three-way ANOVA (Fig. S3h)	$F_{1, 47}$ = 28.073 p < 0.001	$F_{1, 47}$ = 28.506 p < 0.001	$F_{1, 47}$ = 12.460 p < 0.001	$F_{1, 47}$ = 9.126 p = 0.004

Statistical analysis from Fig. S4.

Test	Genotype	Diet	Sex	Interaction
Three-way ANOVA (Fig. S4a)	$F_{1, 46}$ = 64.967; p < 0.001	$F_{1, 46}$ = 5.168; p = 0.029	$F_{1, 46}$ = 12.152; p = 0.001	n.s.
Three-way ANOVA (Fig. S4b)	F1, 46 = 81.867; p < 0.001	n.s.	n.s.	n.s.
Three-way ANOVA (Fig. S4c)	n.s.	F1, 45 = 4.797; p = 0.035	n.s.	n.s.

Statistical analysis from Fig. S5.

Test	Insulin	PTN	Interaction
Two-way ANOVA (Fig. S4a)	$F_{1, 21}$ = 12.595; p < 0.001	n.s.	n.s.
Two-way ANOVA (Fig. S4b)	$F_{1, 22}$ = 11.195; p = 0.003	n.s.	n.s.
Two-way ANOVA (Fig. S4c)	$F_{1, 22}$ = 9.297; p = 0.007	n.s.	n.s.
Two-way ANOVA (Fig. S4d)	$F_{1, 22}$ = 13.453; p = 0.002	n.s.	n.s.
Two-way ANOVA (Fig. S4e)	n.s.	n.s.	n.s.
Two-way ANOVA (Fig. S4f)	n.s.	n.s.	n.s.

Table S4. Schematic summary of the main results of *Ptn* deficiency in males or females on an HFD.

	**Male**				**Female**				**Female vs. Male (as control)**			
	Ptn $^{+/+}$		***Ptn*** $^{-/-}$		***Ptn*** $^{+/+}$		***Ptn*** $^{-/-}$		***Ptn*** $^{+/+}$		***Ptn*** $^{-/-}$	
	**STD**	**HFD**	**STD**	**HFD**	**STD**	**HFD**	**STD**	**HFD**	**STD**	**HFD**	**STD**	**HFD**
**Body weight**		↑↑				↑			↓	↓	↓	↓
**Fasting glucose**		↑↑				↑				↓		
**Insulin**		↑↑↑↑				↑				↓↓		
**Gluconeogenesis**		↑↑	↓	↓		↑↑	↓	↓		↓		
**Insulin resistance**		↑↑↑↑				↑				↓↓		
Ptn		↑	**ND**	**ND**			**ND**	**ND**			**ND**	**ND**
Mdk		↑							↑↑	↑↑	↑↑	↑↑
**ALT**		↑				↑						
**Metainflammation**		↑				↑						
**Pre-albumin**			↑↑↑↑				↑↑↑↑					
**Hepatic steatosis**		↑↑↑↑				↑↑				↓↓		
**Hepatic fibrosis**		↑↑↑↑	↓	↓			↓	↓		↓↓↓↓		

STD: standard diet; HFD: high-fat diet; *Mdk*: Midkine; *Ptn*: Pleiotrophin; ND: no detectable; ALT: alanine aminotransferase.
